# Comparison of diagnostic efficiency of detecting IgG and IgE with immunoassay method in diagnosing ABPA: a meta-analysis

**DOI:** 10.1186/s12890-023-02620-3

**Published:** 2023-10-05

**Authors:** Anlin Liu, Wushu Chen, Yining Wei, Jinkai Liang, Shuhong Liao, Yijun Chen, Yongming Li, Xidong Wang, Weisi Chen, Ye Qiu, Zhengtu Li, Feng Ye

**Affiliations:** 1 State Key Laboratory of Respiratory Disease, National Clinical Research Center for Respiratory Disease, Guangzhou Institute of Respiratory Health, the First Affiliated Hospital of Guangzhou Medical University, Guangzhou, 510120 China; 2 Nanshan School of Guangzhou Medical University, the First Affiliated Hospital of Guangzhou Medical University, 151 Yanjiang Xi Road, Guangzhou, 510120 Guangdong China

**Keywords:** ABPA, Diagnostic accuracy, IgE, IgG, Meta-analysis

## Abstract

**Background:**

Hitherto, the bulk of diagnostic criteria regards *Aspergillus*-specific immunoglobulin E as a key item, and regard IgG as an auxiliary method in diagnose. Nevertheless, there is no conclusive study in summarize the performance of IgG and IgE diagnosing ABPA.

**Methods:**

We conducted a systematic review to identify studies report results of IgE and IgG detection in diagnosing ABPA. QUADAS-2 tool was used to evaluate included studies, and we applied the HSROC model to calculate the pooled sensitivity and specificity. Deeks’ funnel was derived to evaluated the public bias of included studies, and Cochrane Q test and* I*^*2*^ statistic were used to test the heterogeneity.

**Results:**

Eleven studies were included in this study (1127 subjects and 215 for IgE and IgG). Deeks’s test for IgE and IgG were 0.10 and 0.19. The pooled sensitivity and specificity for IgE were 0.83 (95%CI: 0.77, 0.90) and 0.89 (0.83, 0.94), and for IgG were 0.93 (0.87, 0.97) and 0.73 (0.62,0.82), with P value < 0.001. The PLR and NLR for IgE were 7.80 (5.03,12.10) and 0.19 (0.13,0.27), while for IgG were 3.45 (2.40,4.96) and 0.09 (0.05,0.17). The combined diagnostic odds ratio and diagnostic score were 41.49 (26.74,64.36) and3.73 (3.29,4.16) for IgE, respectively, and were 38.42 (19.23,76.79) and 3.65 (2.96,4.34) for IgG.

**Conclusion:**

The sensitivity for IgG diagnosing ABPA is higher than IgE, while the specificity for IgE is higher. IgG might be able to play a more important role in filtering ABPA patients.

**Supplementary Information:**

The online version contains supplementary material available at 10.1186/s12890-023-02620-3.

## Introduction

Allergic bronchopulmonary aspergillosis (ABPA) is a bronchopulmonary allergic inflammatory disease caused by *Aspergillus*, and it is a complex of various immune reactions that leads to immune disorder and it is most commonly combined with asthma and cystic fibrosis [1]. The primary pathogen is *Aspergillus fumigatus* (Af), and a similar disease caused by fungus infection other than *Aspergillus fumigatus* is called allergic bronchopulmonary mycosis (ABPM) [2, 3] The clinical features of ABPA mainly include bronchitis, bronchiectasis, eosinophilia, and Af infection [4]. The pathogenesis of ABPA is related to type I and III allergic reactions [2, 5]. Bronchiectasis, pulmonary cystic changes, fibrosis, and other irreversible changes may occur under repeated attacks of ABPA. Its histological features include mucoid impaction of the bronchi, eosinophilic pneumonia, bronchocentric granulomatosis, and bronchiectasis, but biopsy is not required in the bulk of the patients [3]. Therefore, the early diagnosis of ABPA via immunological methods is very critical and necessary [5].

Hitherto, the published diagnostic criteria for ABPA include Greenberger and Patterson [6], the International Society for Human and Animal Mycology (ISHAM) [7]. The criteria of Rosenberg-Patterson include major criteria and minor criteria, including symptoms, radiological presents, laboratory detections and biopsy, yet not all criteria may not be identified at one time, as some of the feature may only present in specific stage. Meanwhile, the criteria of ISHAM divided the criteria into three aspects, predisposing, obligatory and other criteria. Both criteria have been widely used to diagnose ABPA [8]. The serological examination is of great help in diagnosing and eliminating possible ABPA [5, 9]. The major pathological condition of ABPA is to measure the level of *Aspergillus*-specific immunoglobulin E (IgE) and immunoglobulin G (IgG) antibodies or precipitated antibodies to detect type I and III allergic reactions [5]. The necessary features for diagnosis include elevated total serum IgE > 1000 IU/ml and raised serum IgE antibodies specific for *A. fumigatus*, and the secondary features include raised specific IgG against *A. fumigatus*, eosinophilia, and radiological signs [10].

As mentioned above, the detection of *A. fumigatus* specific IgG and IgE are pivotal for diagnosing ABPA because they reflect type I and III allergic reactions to *Aspergillus* species [2, 5]. There have been studies into the sensitivity and specificity of immunoassay methods detecting IgE and IgG [9, 11]. However, there are differences in the conclusions of these studies. Serological investigations involving rAspf4 and rAspf6 showed that allergen-specific IgE levels against these proteins increased almost exclusively in samples from patients with ABPA [12]. In contrast, de Oliveira et al. [13] showed that the determination of serum IgE against recombinant *A. fumigatus* allergen was not helpful in diagnosing ABPA or detecting sensitization to fungus.

Considering that there is no conclusive study on the specificity and sensitivity of detection of *A. fumigatus*-specific IgG and IgE performed concurrently in the same set of patients, we aimed to find whether detection of *A. fumigatus* specific IgG or IgE by immunoassay-methods would perform better in diagnosing ABPA in this systematic review.

## Methods

### Study design

We conducted this systematic review and meta-analysis according to the Preferred Reporting Items for Systematic Reviews and Meta-Analyses for diagnostic test accuracy (PRISMA-DTA) statement [14]. Our study did not require ethics committee approval. Our study has been registered in the PROSPERO database (CRD42023390030).

### Search strategy

Two investigators (AL and WS) independently searched the EMBASE and PubMed databases for records (until July 30). The search strategy includes terms including “ABPA”, “IgG”, and “Immunoassay”, and the detailed strategy is shown in Supplementary Methods 1.

### Inclusion and exclusion criteria

We included studies meeting the following criteria:

- studies describing immunoassay methods for detecting both IgE (total IgE or/and A. fumigatus- specific IgE antibodies) and A. fumigatus-specific IgG antibodies for diagnosing ABPA;
- studies reporting the diagnostic accuracy values of both techniques or allowing the calculation of sensitivity from the study observations;
- the studies had a control group and an experimental group.

Studies that met the following criteria would be excluded:

- studies including case reports, abstracts, comments, editorials, and reviews;
- studies only describing either the IgE or IgG for diagnosing ABPA;
- studies published in a language other than English.

### Study outcome

The primary outcome of our study is the sensitivity and specificity of detecting IgG and IgE with the immunoassay method in diagnosing ABPA. We used various methods to evaluate the diagnostic performance of IgG and IgE, including the HSROC model, random-effect model, and diagnostic odds ratios.

### Initial review of studies

**Fig. 1:**
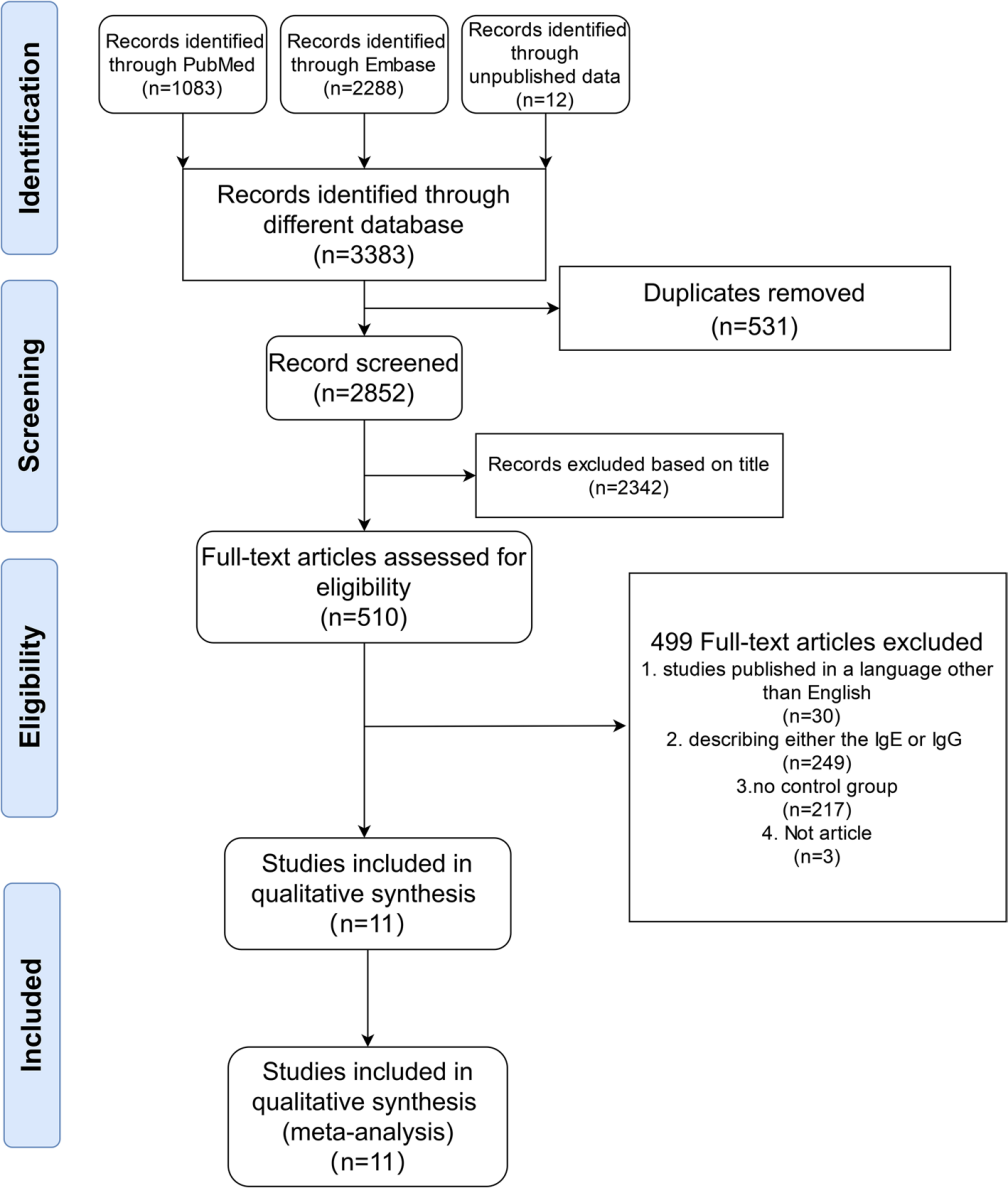
Preferred Reporting Items for Systematic Reviews and Meta-Analysis (PRISMA) flow diagram of the search results and article exclusion details

A total of 3383 studies were obtained via PubMed and Embase, and unpublished data in our institution, which identify 510 possible studies in the first stage. All articles were imported into a file manager (Endnote 20, Clarivate Analytics, Philadelphia, USA), then two authors (AL and WS) screened the files by reviewing the title and abstract after dropping the duplicates. Any disagreement would be handed to a third author (YQ) and reached consensus after discussion. In subsequent, authors (JL and YW) would conduct the second screening based on the full text, and the inclusion and exclusion criteria were applied. The Preferred Reporting Items for Systematic Reviews and Meta-Analysis (PRISMA) flow diagram of the search results and article exclusion details are shown in Fig. 1.

### Data extraction

Two authors (AL and WC) independently extracted data using a preapproved electric form by all authors. Retrieved data included a few dimensions:

- publication information (author, publication year, country);
- study design (case–control study, cohort study or others);
- the inclusion and exclusion criteria for the study and the number of studies;
- the type of control population included;
- the criteria for diagnosing ABPA;
- the detailed detecting methods (ELISA, ImmunoCAP or others).

Any difference would be submitted to another author, QY, to resolve. Studies with high quality and detailed sensitivity and specificity for both IgE and IgG would be selected.

### Quality assessment

We use the QUADAS-2 to measure the quality of included studies, which brings more transparency to bias assessment and diagnostic laboratory evaluation [15]. RevMan (Review Manager, version 5.4, Copenhagen: The Nordic Cochrane Centre, The Cochrane Collaboration, 2014) was used to evaluate the publication bias. Two authors (YW and JL) assessed included studies, and conflicts were solved with the third author (AL). Items in the tool were rated as low, high and unclear based on several dimensions: patient selection, index test, reference standard and flow and timing.

### Statistical analysis

The Rutter and Gatsonis hierarchical model has been proved to be effective in diagnostic meta-analysis, and has been widely used to provide a summary ROC curve, as different ranges of thresholds for immunoassay were used by different investigators [16]. The main outcomes were the summarized sensitivity and specificity, diagnostic likelihood ratio (DLR) positive and DLR negative, and diagnostic odds ratio and diagnostic score for IgG and IgE diagnosing ABPA with immunoassay method. The 95% confidence intervals were calculated with Clopper-Pearson method. The pooled sensitivity and specificity were calculated using the bivariate random-effects model, and we also used receiver operating characteristic (ROC) space and the hierarchical summary receiver operating characteristic (HSROC) model independently for comparing the accuracy for IgG and IgE in diagnosing ABPA [17, 18]. Besides, sensitivity analysis was conducted to examine the stability of the finding. It’s noteworthy that if the value for beta (the shape parameter for curve) was closed to zero, the plot was more likely to have no association between test accuracy and test threshold. The heterogeneity of the main outcomes was assessed with Cochran’s Q statistic, with *P* < 0.10 denoting heterogeneity, while the I [2] statistic was also used, whose values greater than 50% were considered to denote heterogeneity. To evaluate the publication bias of included studies, Deek’s test was used [19], with *P* < 0.01 indicating significant public bias.

We used Stata17 (Statistics and Data Science, Stata Corp LLC, College Station, TX, USA) to conducted all of the statistical analyses.

## Results

After the initial search, we found 3383 articles (1083 for PubMed and 2288 for Embase, and 12 for unpublished data). After screening the abstracts and titles and evaluating the eligibility for research based on the inclusion and exclusion criteria, eight manuscripts were included in our study, with 1127 subjects and 215 for IgE and IgG [5, 9, 20–25], respectively. Due to the differences in various dimensions of included studies, for example, the exact method for immunoassay, the different cut-off values, and different antigens, the studies were heterogeneous.

### Details of included studies

All included studies were case–control studies, among which 4 used ELISA [20–22], 4 used ImmunoCAP [5, 9, 20, 23] and 3 used other methods [20, 24, 25]. Besides, two studies establish control based on healthy subjects and subjects with diseases [5, 9]. For the diagnostic criteria of ABPA, s studies used the International Society for Human and Animal Mycology criteria (2013) [9, 20, 21, 23, 24], 2 used Rosenburg-Patterson criteria [20, 25], 1 for Patterson criteria [5], and 1 for Nelson’s criteria [22]. The details of included studies were shown in supplementary materials (Supplementary Table 1).

### Quality assessment

Quality assessment was conducted with the QUADAS-2 tool, and the detailed is showed in supplementary materials (Supplementary Fig. 1), which met the requirement of quality-control guidelines. The results showed that though 7 studies were rated high risk of bias in “patient selection”, the bulk of risk is low.

### Diagnostic accuracy for each antibody

**Fig. 2:**
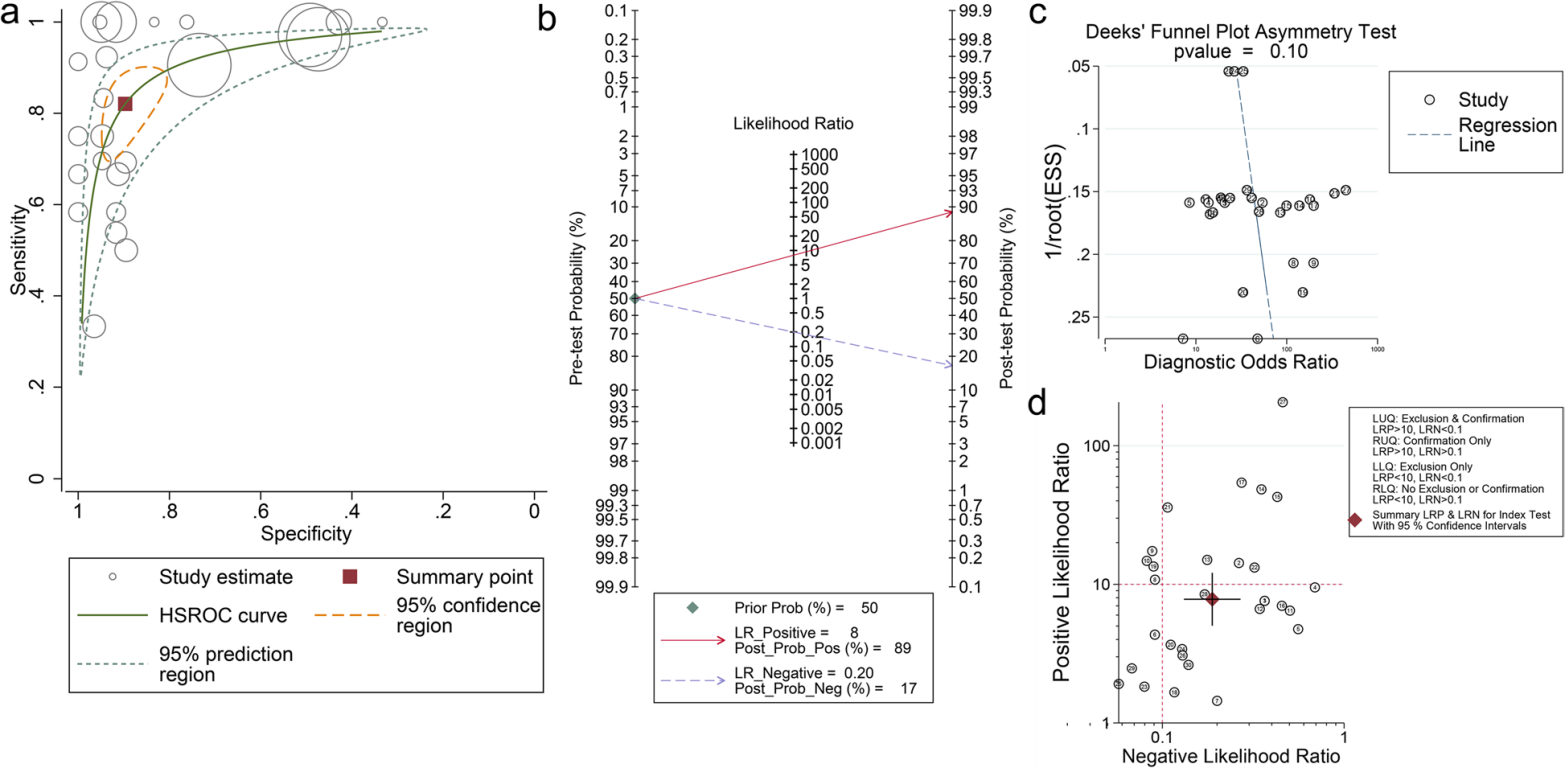
**a** HSROC figure for detecting IgE in diagnosing ABPA. The pooled sensitivity for IgE diagnosing ABPA is 0.83 (95%CI: 0.76, 0.89), and specificity is 0.89(0.83, 0.94). The solid cube indicates the summary diagnostic accuracy points, while the orange dashed lines represent 95% confidence regions around these summary estimates and green dashed lines represent the 95% prediction region; **b** Fagan plot for included studies; **c** Deeks’ funnel plot for included studies, the *p*-value for Deek’s test is 0.10, which shows no significant bias; **d** distribution scatter diagram of the likelihood ratio (LR + /LR-) of each study and combined estimated value

**Fig. 3:**
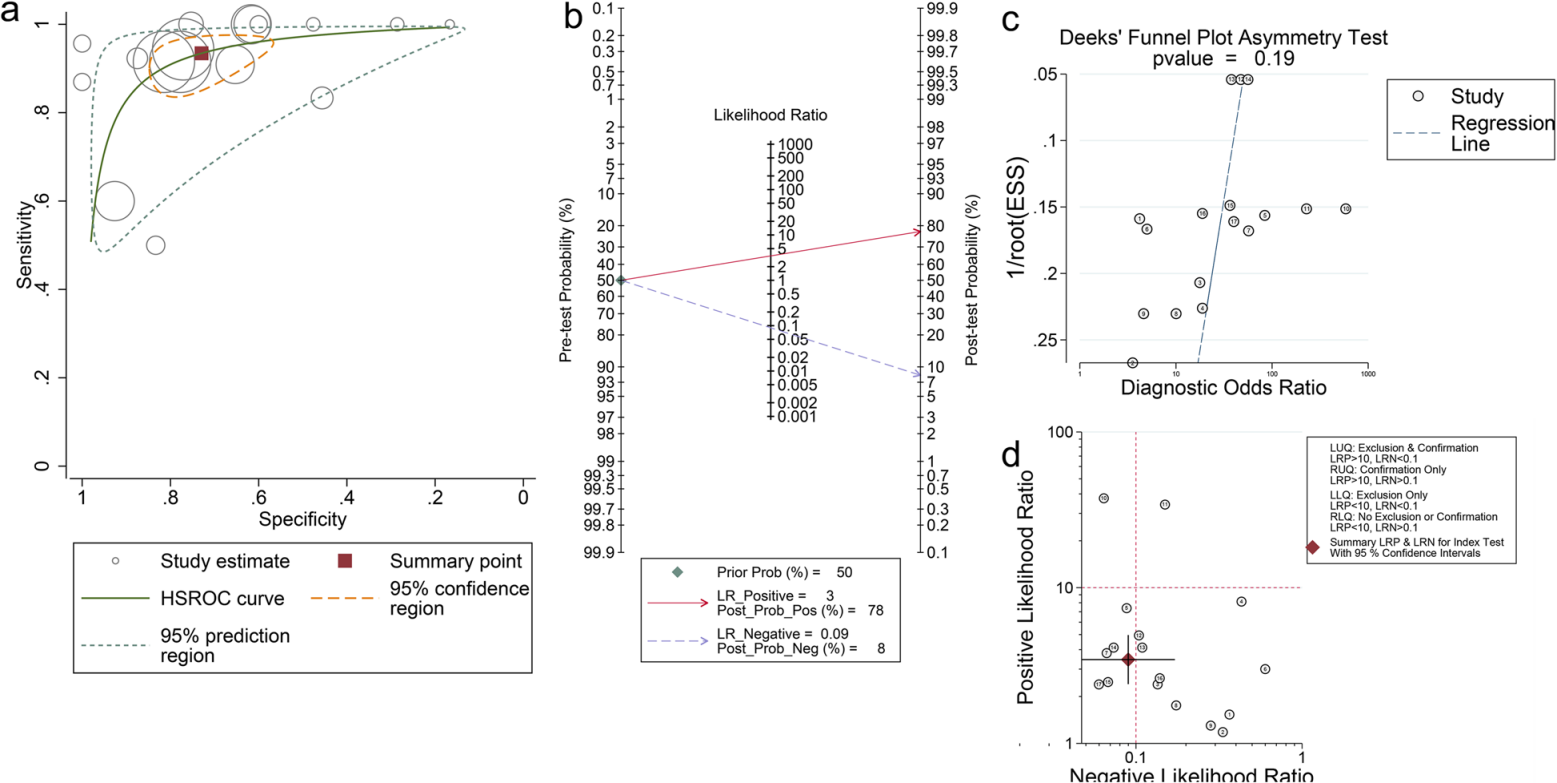
**a**. HSROC figure for detecting IgG in diagnosing ABPA. The sensitivity for IgG diagnosing ABPA is 0.93(0.87, 0.97), and specificity is 0.73 (0.62,0.82). The solid cube indicates the summary diagnostic accuracy points, while the orange dashed lines represent 95% confidence regions around these summary estimates and green dashed lines represent the 95% prediction region; **b** Fagan plot for included studies; **c** Deeks’ funnel plot for included studies. The *p*-value for Deek’s test is 0.19, which shows no significant bias; **d** distribution scatter diagram of the likelihood ratio (LR + /LR-) of each study and combined estimated value

The HSROC curve showed that compared to the curve of IgE (Fig. 2a), the curve for IgG was closer to zero point (Fig. 3a), and the pooled sensitivity for IgG is 0.93(95%CI: 0.87, 0.97), which is higher than IgE: 0.83 (95%CI: 0.76, 0.89). However, the pooled specificity for IgE and IgG were 0.89 (0.83 0.94) and 0.73 (0.61,0.82), respectively. The summary performance of IgE and IgG in diagnosing ABPA is shown in Supplementary Table 2. The forest plots for pooled sensitivity and specificity for IgE and IgG diagnosing ABPA were shown in Supplementary Figs. 2 and 4.

The bulk of the pooled estimates with a 95% confidence interval was located in the lower right quadrant of the scatter plot of the likelihood ratios (Figs. 2d, and 3d), suggesting the combined accuracy of IgG and IgE diagnosing ABPA is low.

### Positive likelihood ratio and negative likelihood ratio (PLR and NLR)

#### Fagan nomogram analysis

Fagan plots were conducted in our studies. A 50% predicted probability was assessed respectively for IgE and IgG to simulate a clinical situation. For IgE, the post-probability for positive was 89%, and for negative, it was 16% (Fig. 2b), while the post-probability for positive was 78%, and for negative was 8% (Fig. 3b). The likelihood ratio_positive (LR_P) was 8 and 3 for IgE and IgG, and the likelihood ratio_negative (LR_N) was 0.19 and 0.09. The results indicated that the IgE test enhanced the diagnosis accuracy, and the IgG test would identify more negative patients.

#### Forest plot for PLR and NLR

The pooled positive likelihood ratio and negative likelihood ratio for IgE were 7.80 (5.03, 12.10) and 0.19 (0.13, 0.27) (Supplementary Fig. 3), and for IgG were 3.45 (2.4, 4.96), and 0.09 (0.05, 0.17) (Supplementary Fig. 6).

#### Diagnostic odds ratios and diagnostic scores

The combined diagnostic odds ratio and diagnostic score were 41.49 (26.74, 64.36), and 3.73(3.29–4.16) for IgE, respectively (Supplementary Fig. 4), and were 38.42 (19.23, 76.79) and 3.65 (2.96,4.34) for IgG, respectively (Supplementary Fig. 7).

#### Bias assessment and sensitivity analysis

We conducted Deek’s test to evaluate the publication bias, and P value < 0.01 was considered significant publication bias. P values for IgE and IgG were 0.10 and 0.19 (Fig. 2c and Fig. 3c), respectively. We conducted the sensitivity analysis to examine the stability of finding, and the results showed that the heterogeneity did not comes from single research (Supplementary Fig. 8a, b).

## Discussion

Diagnostic meta-analysis requires careful assessment of included studies. As a widely acknowledged tool to evaluate the quality of diagnostic meta-analysis [15], we used QUADAS-2 tool to control the quality of this research.,ABPA is a mysterious issue, which is an inflammatory diseased induced by infection, and it is a type I and type III hypersensitivity-mediated allergic reaction to *Aspergillus*, and various inflammatory mediators were induced by *Aspergillus* conidia, including interleukin (IL)-4, IL-5, IL-13 and other cytokines, and inflammatory cells such as eosinophils would be also stimulated [26]. To diagnose this disease correctly, plenty of different criteria had been proposed, and played a critical role in clinical practice, nevertheless, it is hard for clinical practitioners to reach agreements [27]. Previous study had proved that immunoassay methods is more sensitive than immunoprecipitation, and enjoys a similar specificity [11]. In this study, we identified that the sensitivity for detecting IgG for diagnosing ABPA with immunoassay method was higher than IgE, while the specificity for IgE was more specific, which might allow clinicians to make better clinical choice.

IgE had been regarded as a crucial criterion in diagnosing ABPA, especially an elevated level of serum *A. fumigatus*-specific IgE, which is regarded the most sensitivity investigation methods in diagnosing ABPA [3, 7, 28]. Previous study has proved that In a large prospective study, the sensitivity and specificity of *A. fumigatus*-specific IgE were found to be 100% and 70%, respectively, in the diagnosis of ABPA [29]. In contrast, our study indicated that the pooled sensitivity of IgE is0.83 (0.76,0.89), and we owe this to the various level of baseline serum total IgE in different country [30].

Meanwhile, the importance of IgG seemed to be underestimated. A cohort study suggested that *A. fumigatus*-specific IgG is valuable in diagnosing ABPA, whose sensitivity is 89% and specificity is 100% [31]. In our study, the pooled sensitivity for IgG is 0.93(0.87,0.97) and the pooled specificity is 0.73(0.61,0.82), which indicated that the values of IgG deserve more emphasis, especially in filtering possible ABPA patients. Nevertheless, there are differences between our studies and studies published before, we believed that the reasons for the variances might relate to the different races, equipment, and most importantly, different method for immunoassay. *A. fumigatus*-specific IgG detected using double gel diffusion technique has a sensitivity of only 27% in the diagnosis of ABPA while the commercial enzyme immunoassay methods for measuring *A. fumigatus*-specific IgG have a sensitivity exceeding 90% [31, 32]. We believed that the reason for a lower specificity for IgG diagnosing APBA may relate to its advantages in diagnosing and monitoring chronic pulmonary Aspergillosis (CPM). According to several cohort studies, IgG played an essential role in diagnosing chronic ABPA, and had a higher value than *A. fumigatus*-specific IgE, which strongly indicate possible active CPM [33, 34]. Therefore, further research and clinical trial is vital for clarifying the relationship.

Though we found interesting results in our study, it is noteworthy that though serum tests of IgE and IgG is important in diagnosing ABPA, the diagnose requires a more comprehensive examination. For example, the level of serum total IgE may lower in different country [30], and put too much emphasis on serum test may lead to mis diagnose of ABPA. Therefore, a wholesome examination is the key to diagnose ABPA correctly. The founding of bronchocentric granulomatosis, noninvasive fungal hyphae, and mucoid impaction of bronchi in tissue sample is an essential criterion in diagnosis of ABPA [35]; radiological presentations including mucus plug, central bronchiectasis and fleeting opacities play an important role in diagnosis [36]; methods of finding pathogens such as fungal culture are a key focus and difficulty in diagnosis. New diagnostic techniques such as mNGS can better identify pathogens and help make an early and accurate diagnosis [37, 38]. In conclusion, a solid diagnosis of ABPA requires a holistic approach, a deep understanding of the diagnostic criteria and a comprehensive judgement of the patient's condition. Clinicians should be aware that ABPA is possible if patients have significant gasping for breath, with positive IgG and negative IgE, and more examinations including lung function test should be considered.

This study suffers from serval limitations. First of all, as shown in Supplementary Fig. 1, the risk of bias and applicability of patient selection was high. The reason for this issue was that included studies were all unblind-cast-control study, and there was risk in patient selection bias. Besides, the diagnostic criteria for ABPA were different in each study, which may also lead to patient selection bias. Moreover, as immunoassay method is a quantitative method, the thresholds for different studies were different, and the sensitivity and specificity might be estimated higher or lower. Last but not least, the included studies with different designs for control group. The control group in 2 studies contained both healthy and patients with Aspergillosis [5, 9], which may lead to higher possible bias.

## Conclusion

The sensitivity for IgG in diagnosing ABPA is higher than IgE, and the specificity of IgE is higher than IgG. The value of IgG in diagnosing ABPA should not be underestimated, and it may play a more important role in filtering possible ABPA patients.

### Supplementary Information

**Additional file 1:** **Supplementary Table 1.** Detailed information of included studies. **Supplementary Table 2.** Summary performance for IgE and IgG in diagnosing ABPA. **Supplementary Figure 1.** Risk of bias and applicability concerns summary. **Supplementary Figure 2.** Forest plot of pooled sensitivity and specificity of the included articles (*n* = 12). **Supplementary Figure 3.** Forest plot of positive likelihood ratio and negative likelihood ratio of the included articles (*n* = 12). **Supplementary Figure 4.** Forest plot of the diagnostic score and diagnostic odds ratio of the included articles (*n* = 12). **Supplementary Figure 5.** Forest plot of pooled sensitivity and specificity of the included articles (*n* = 12). **Supplementary Figure 6.** Forest plot of positive likelihood ratio and negative likelihood ratio of the included articles (*n* = 12). **Supplementary Figure 7.** Forest plot of the diagnostic score and diagnostic odds ratio of the included articles (*n* = 12). **Supplementary Figure 8.** Sensitivity analysis of IgE and IgG (*n* = 12).

## Data Availability

The datasets generated during this study can be available from the corresponding author on reasonable request.

## Abbreviation

ABPA: Allergic bronchopulmonary aspergillosis
ABPM: Allergic bronchopulmonary mycosis
Af: *Aspergillus fumigatus*
DLR: Diagnostic likelihood ratio
ELISA: Enzyme linked immunosorbent assay
HSROC: Hierarchical summary receiver operating characteristic model
IgG: Immunoglobulin G
IgE: Immunoglobulin E
LR_N: Likelihood ratio_negative
LR_P: Likelihood ratio_positive
PRISMA-DTA: Preferred Reporting Items for Systematic Reviews and Meta-Analyses for diagnostic test accuracy
ROC: Receiver operating characteristic
