# Supplementary material for: Comparison of diagnostic efficiency of detecting IgG and IgE with immunoassay method in diagnosing ABPA: a meta-analysis
Source: BMC Pulm Med. 2023 Oct 5;23:374. doi: 10.1186/s12890-023-02620-3 (PMC10557217; doi:10.1186/s12890-023-02620-3)
Supplement: Supplementary file 1 — Additional file 1: Supplementary Table 1. Detailed information of included studies. Supplementary Table 2. Summary performance for IgE and IgG in diagnosing ABPA. Supplementary Figure 1. Risk of bias and applicability concerns summary. Supplementary Figure 2. Forest plot of pooled sensitivity and specificity of the included articles (n = 12). Supplementary Figure 3. Forest plot of positive likelihood ratio and negative likelihood ratio of the included articles (n = 12). Supplementary Figure 4. Forest plot of the diagnostic score and diagnostic odds ratio of the included articles (n = 12). Supplementary Figure 5. Forest plot of pooled sensitivity and specificity of the included articles (n = 12). Supplementary Figure 6. Forest plot of positive likelihood ratio and negative likelihood ratio of the included articles (n = 12). Supplementary Figure 7. Forest plot of the diagnostic score and diagnostic odds ratio of the included articles (n = 12). Supplementary Figure 8. Sensitivity analysis of IgE and IgG (n = 12). [file 12890_2023_2620_MOESM1_ESM.zip › 12890_2023_2620_MOESM10_ESM.docx]

**Supplementary Materials for**

**Comparison of Diagnostic Efficiency of Detecting IgG and IgE with Immunoassay Method in Diagnosing ABPA: A Meta-analysis**

Content

[Supplementary Methods 4](#_Toc143460666)

[Supplementary Method 1: Search strategy 4](#_Toc143460667)

[Supplementary Tables 5](#_Toc143460668)

[Supplementary Table 1: Detailed information of included studies 5](#_Toc143460669)

[Supplementary Table 2: Summary performance for IgE and IgG in diagnosing ABPA 7](#_Toc143460670)

[Supplementary Figures 8](#_Toc143460671)

[Supplementary Figure 1: Risk of bias and applicability concerns summary 8](#_Toc143460672)

[Supplementary Figure 2: Forest plot of pooled sensitivity and specificity of the included articles (n = 12) 9](#_Toc143460673)

[Supplementary Figure 3: Forest plot of positive likelihood ratio and negative likelihood ratio of the included articles (n = 12) 10](#_Toc143460674)

[Supplementary Figure 4: Forest plot of the diagnostic score and diagnostic odds ratio of the included articles (n = 12) 11](#_Toc143460675)

[Supplementary Figure 5: Forest plot of pooled sensitivity and specificity of the included articles (n = 12) 12](#_Toc143460676)

[Supplementary Figure 6: Forest plot of positive likelihood ratio and negative likelihood ratio of the included articles (n = 12) 13](#_Toc143460678)

[Supplementary Figure 7: Forest plot of the diagnostic score and diagnostic odds ratio of the included articles (n = 12) 14](#_Toc143460680)

[Supplementary Figure 8: Sensitivity analysis of IgE and IgG (n = 12) 15](#_Toc143460682)

[PRISMA Checklist for Comparison of Diagnostic Efficiency of Detecting IgG and IgE with Immunoassay Method in Diagnosing ABPA: A Meta-analysis 16](#_Toc143460683)

[Reference: 20](#_Toc143460684)

# Supplementary Methods

# Supplementary Method 1: Search strategy

#1：

allergic bronchopulmonary aspergillosis OR ABPA OR abpa OR allergic bronchopulmonary mycosis OR fungal sensitization OR fungal allergy OR mould allergy OR fungal asthma OR ABPM OR abpm

#2:

precipitin OR precipitins OR gel diffusion OR immunoelectrophoretic OR cie OR immunoassay OR EIA OR ELISA OR enzyme linked immunosorbent assay OR serological assay OR immunoglobulin OR IgG OR antibody assay OR RIA OR IgE

# Supplementary Tables

# Supplementary Table 1: Detailed information of included studies

| Author(date) | Country | Study design | Number of subjects with ABPA | Numbers of controls | Immunoassay test method | Diagnostic criteria for ABPA | Threshold of detection | Antigens for immunoassay |
| --- | --- | --- | --- | --- | --- | --- | --- | --- |
| Kuwabara et al (2019)^1^ | Japan | case-control study | 12 | D=6  H=41 | ImmunoCAP | Patterson criteria | IgE: 0.35UA/mL;  IgG: 26.9mgA/L. | ND |
| Braun. et al (2007)^2^ | USA | case-control study | 6 | 6 | ELISA | Rosenburg-Patterson criteria | ≥0.8 | In house |
| Hsiao et al (2022)^3^ | Taiwan, China | case-control study | 5 | D =99  H=118 | ImmunoCAP | ISHAM (2013) criteria | 0.35 KUA/L | CA |
| Fricker-Hidalgo et al (2010)^4^ | France | case-control study | 13 | 48 | ELISA | ISHAM (2013) criteria | sIgE: >0.1 KAU/L;  total IgE:>500 kIU/L  IgG =0.75 | CA |
| Latzin et al (2007)^5^ | Switzerland | case-control study | 12 | 36 | ELISA | Nelson’s criteria | IgE: 500 IU/mL)  sIgE: 17.5 IU/mL IgG: 20 kU/L | CA |
| Maleki et al (2020)^6^ | Iran | case-control study | 9 | 77 | ImmunoCAP | ISHAM (2013) criteria | sIgEAf ：0.35KAU/L  sIgGAf ：12U/ml | CA |
| Barrera et al (2015)^7^ | France | case-control study | 5 | 21 | ImmunoCAP, DELFIA and WB | ISHAM (2013) criteria | IgE ＞0.35 kIU/L  total IgE level ＞500 IU/ml | CA |
| Wang et al (1977)^8^ | USA | case-control study | 23 | 19 | PTRIA and LARIA | Rosenburg-Patterson criteria | NA: | ND |
| Saxena et al (2020)^9^ | India | case-control study | 106 | 437 | the fluorescent enzyme immunoassay technology | Rosenburg-Patterson criteria/ ISHAM (2013) criteria | Total IgE (>417IU/mL, >500IU/mL, and >1000IU/mL)  IgG (>27mg/) | ND |
| Mortezaee et al.. (2021)^10^ | Iran | case-control study | 11 | 189 | PCR | Rosenburg-Patterson criteria/ISHAM criteria | sIgEAf >0.35 KUA/L and sIgGAf values > 12 U/ml  Total IgE > 1000 IU/ml and > 417 IU/ml. | CA |
| Brummund et al (1986)^11^ | USA | case-control study | 13 | 30 | ELISA | Rosenburg-Patterson criteria | sIgEAf >0.21IUA/L a | ND |

D: subjects with disease; H: healthy subjects; ELISA: enzyme linked immunosorbent assay; DELFIA: dissociation enhanced lauthanide fluoroimmunoassay; WB: western blot; PTRIA: polystyrene tube radioimmunoassay; LARIA: radioimmunoassay by Aspergillus fumigatus antigen labeled with iodine-125; ISHAM: International Society for Human and Animal Mycology NA: not available; ND: not defined; CA: commercially available.

# Supplementary Table 2: Summary performance for IgE and IgG in diagnosing ABPA

|  | IgE | IgG |
| --- | --- | --- |
| Sensitivity | 0.83 (0.76, 0.89) | 0.93 (0.87, 0.97) |
| Specificity | 0.89 (0.83, 0.94) | 0.73 (0.61, 0.82) |
| Positive Likelihood Ratio | 7.80(5.03, 12.10) | 3.45 (2.40, 4.96) |
| Negative Likelihood Ratio | 0.19 (0.13, 0.27) | 0.09 (0.05, 0.17) |
| Diagnostic Odds Ratio | 41.49(26.74, 64.36) | 38.42 (19.23, 76/79) |

# Supplementary Figures

# Supplementary Figure 1: Risk of bias and applicability concerns summary


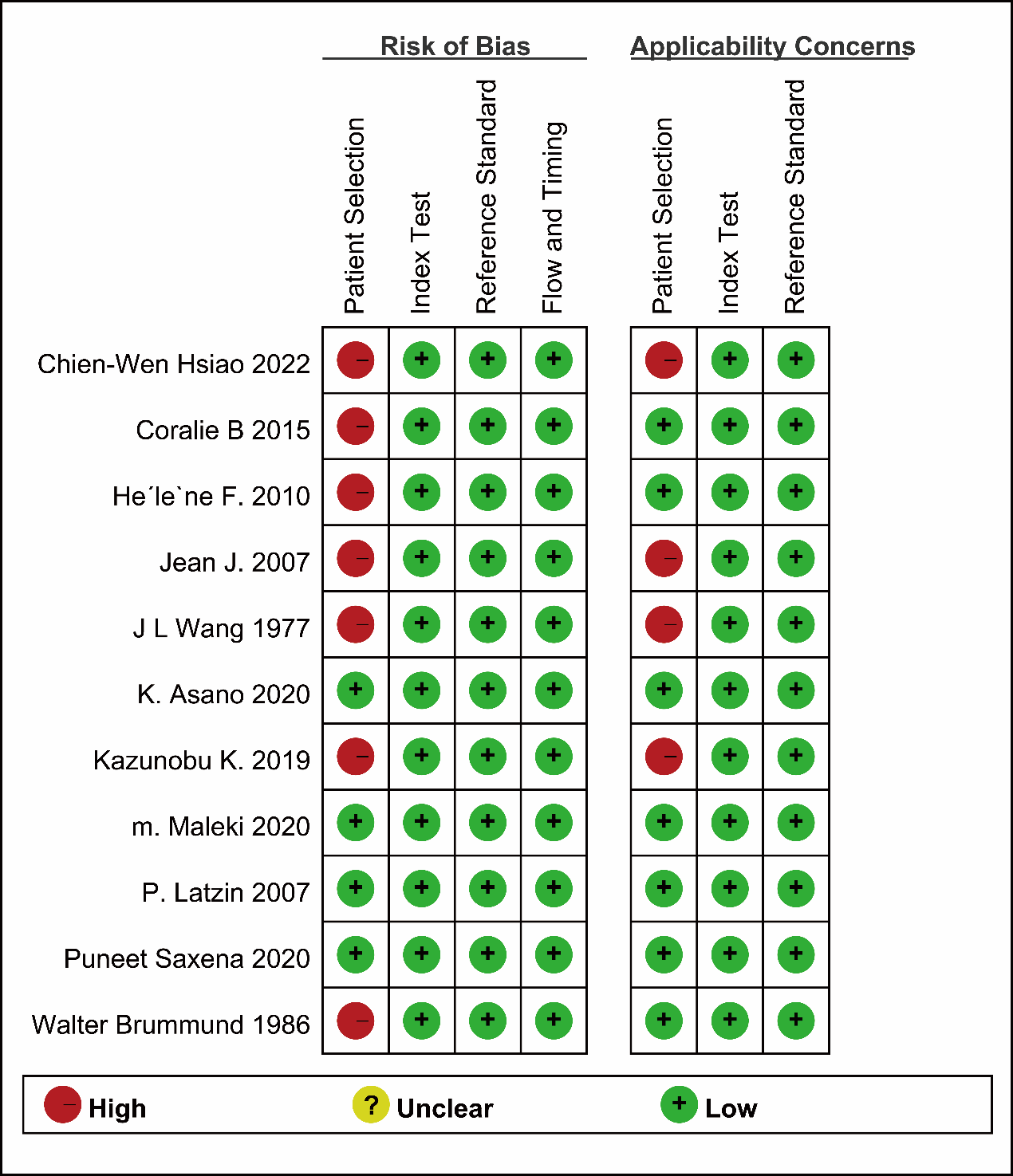


Risk of bias and applicability concerns summary: review authors' judgements about each domain for each included study. Included studies were assessed with Quality Assessment of Diagnostic Accuracy Studies-2 (QUADAS-2).

# **Supplementary Figure 2:** Forest plot of pooled sensitivity and specificity of the included articles (n = 12)


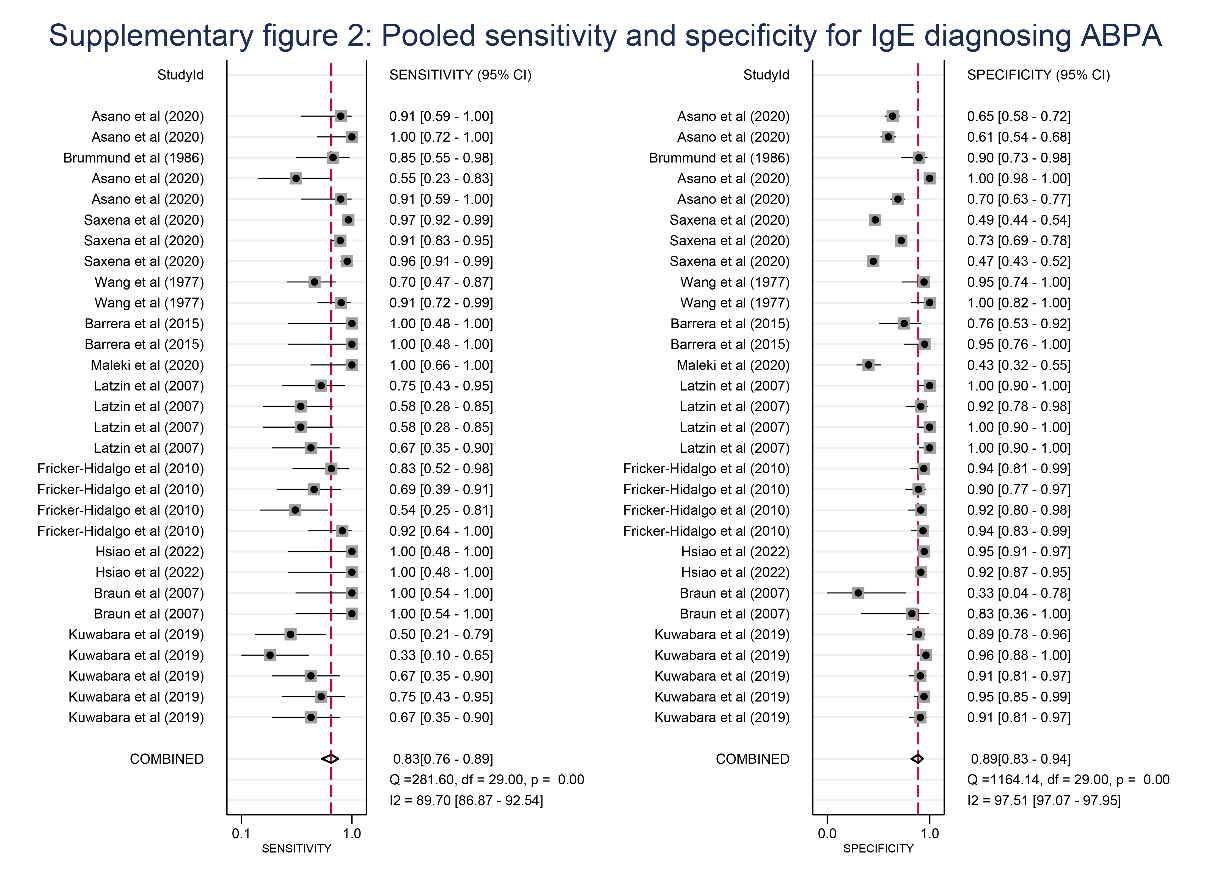


IgE, Immunoglobulin E; ABPA, allergic bronchopulmonary aspergillosis; CI, confidence interval; The pooled sensitivity and specificity for all data of IgE was 0.83 (0.76-0.89) and 0.89 (0.83-0.94).

# Supplementary Figure 3: Forest plot of positive likelihood ratio and negative likelihood ratio of the included articles (n = 12)
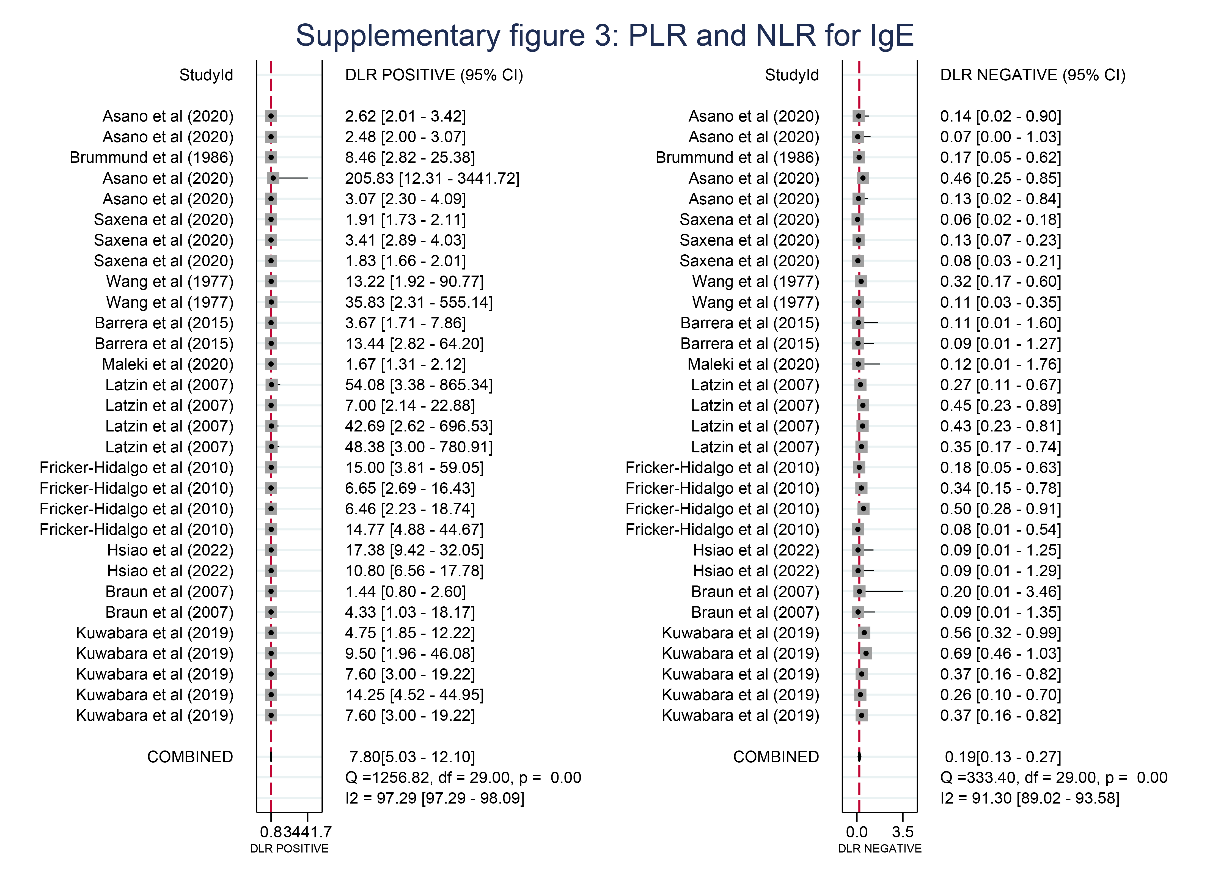


IgE, Immunoglobulin E; PLR: positive likelihood ratio; NLR: negative likelihood ratio; CI, confidence interval; The positive likelihood ratio and negative likelihood ratio for all data of IgE were 7.80(5.03-12.10) and 0.19(0.13-0.27).

# Supplementary Figure 4: Forest plot of the diagnostic score and diagnostic odds ratio of the included articles (n = 12)


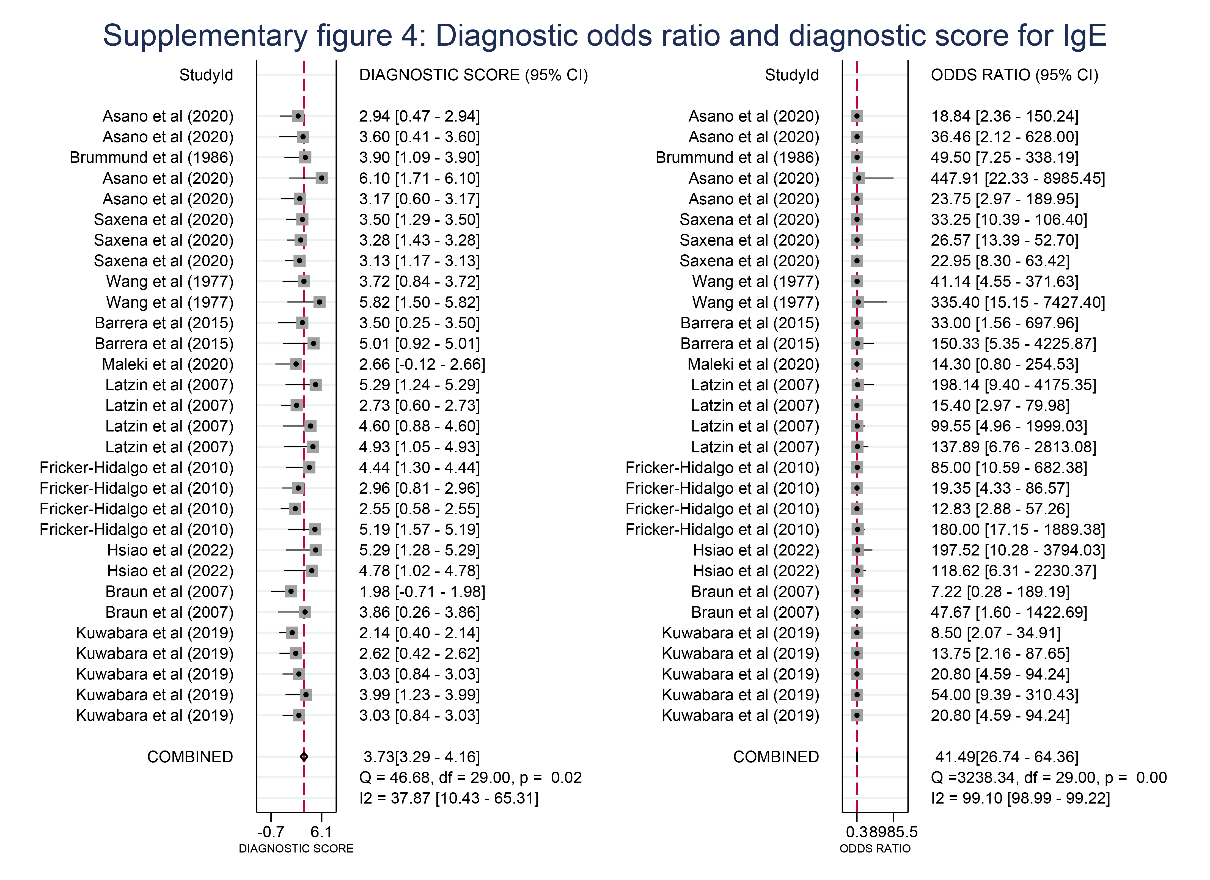


IgE, Immunoglobulin E; CI, confidence interval; The diagnostic score and diagnostic odds ratio for all data of IgE was 3.73(3.29-4.18) and 41.49(26.74-64.36).

# Supplementary Figure 5: Forest plot of pooled sensitivity and specificity of the included articles (n = 12)


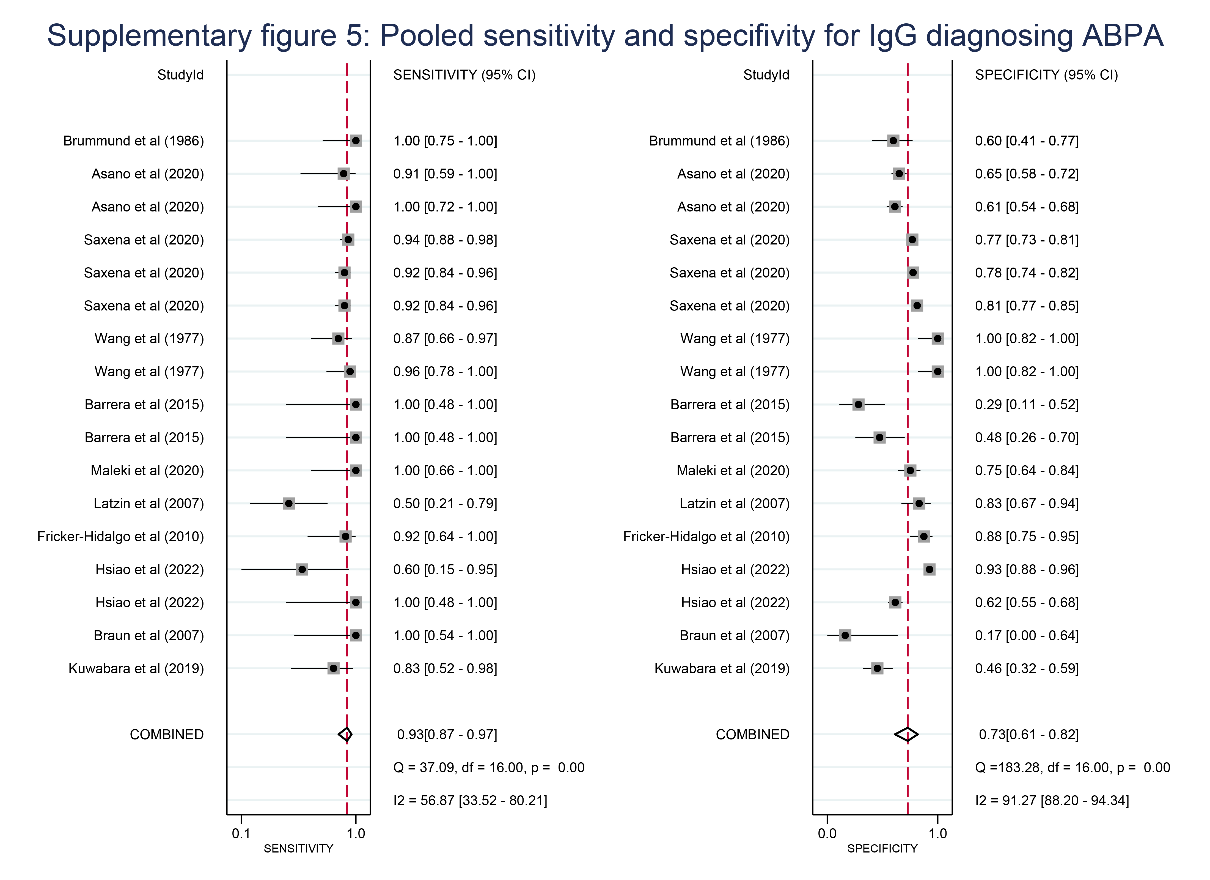


IgG, Immunoglobulin G; ABPA, allergic bronchopulmonary aspergillosis; CI, confidence interval; The pooled sensitivity and specificity for all data of IgG were 0.93(0.87, 0.97) and 0.73(0.61-0.82).

# Supplementary Figure 6: Forest plot of positive likelihood ratio and negative likelihood ratio of the included articles (n = 12)


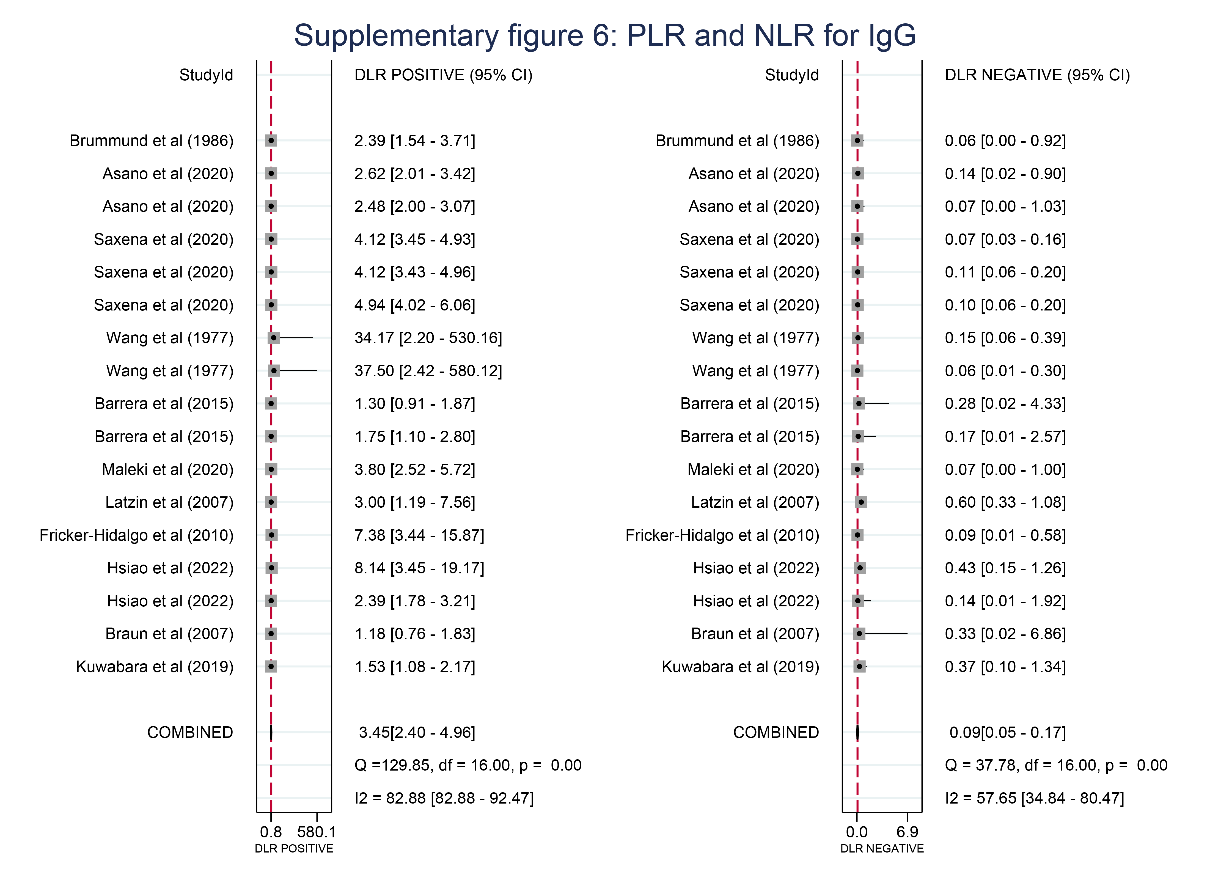


IgG, Immunoglobulin G; PLR: positive likelihood ratio; NLR: negative likelihood ratio; CI, confidence interval; The positive likelihood ratio and negative likelihood ratio for all data of IgG were 3.45(2.40-4.96) and 0.09(0.05-0.17).

# Supplementary Figure 7: Forest plot of the diagnostic score and diagnostic odds ratio of the included articles (n = 12)


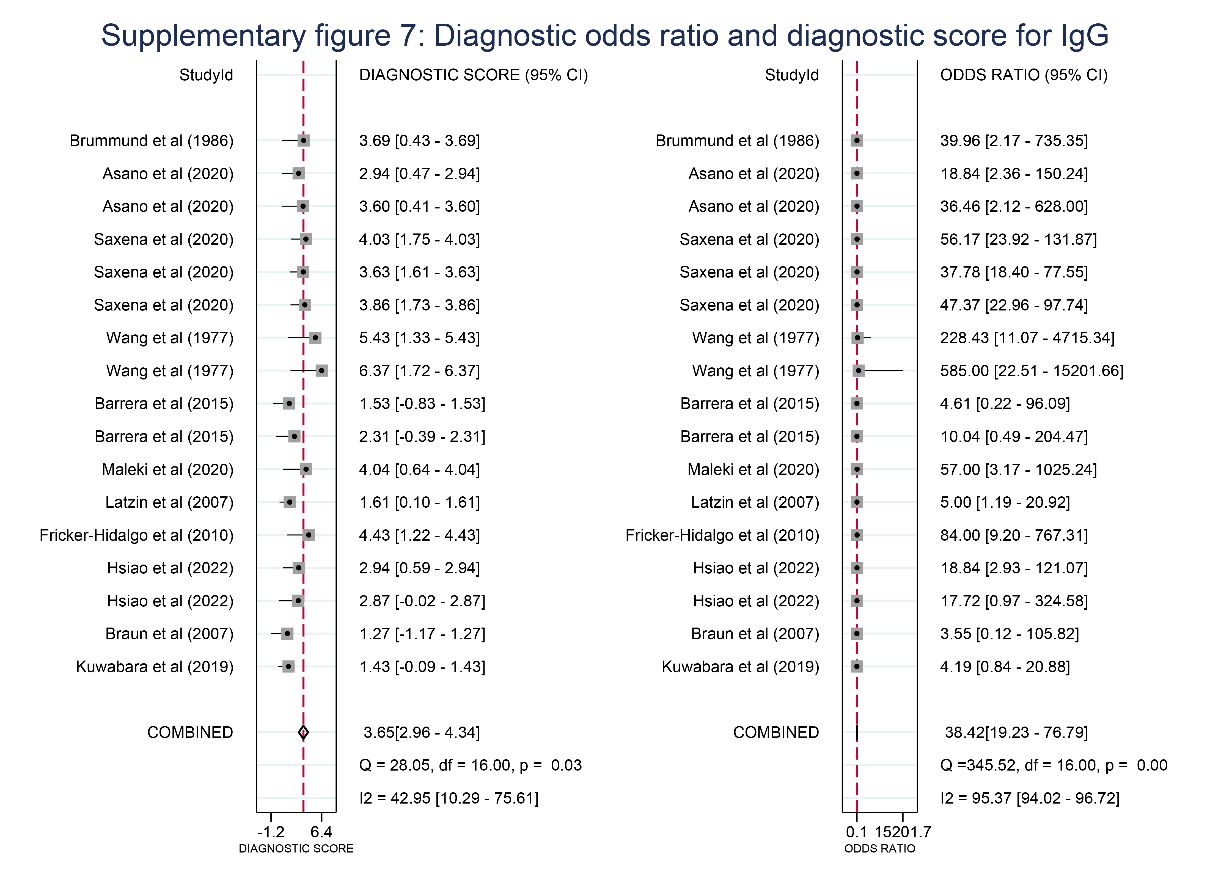


IgG, Immunoglobulin G; CI, confidence interval; The diagnostic score and diagnostic odds ratio for all data of IgG was 3.65(2.96-4.34) and 38.42(19.23-76.79).

# Supplementary Figure 8: Sensitivity analysis of IgE and IgG (n = 12)

Supplementary figure 8a

Supplementary figure 8b

a. Sensitivity analysis of IgE diagnosing ABPA; b. Sensitivity analysis of IgG diagnosing ABPA

# PRISMA Checklist for Comparison of Diagnostic Efficiency of Detecting IgG and IgE with Immunoassay Method in Diagnosing ABPA: A Meta-analysis

| **Section and Topic** | **Item #** | **Checklist item** | **Location where item is reported** |
| --- | --- | --- | --- |
| **TITLE** | | |  |
| Title | 1 | Identify the report as a systematic review. | 1 |
| **ABSTRACT** | | |  |
| Abstract | 2 | See the PRISMA 2020 for Abstracts checklist. | 3 |
| **INTRODUCTION** | | |  |
| Rationale | 3 | Describe the rationale for the review in the context of existing knowledge. | 4 |
| Objectives | 4 | Provide an explicit statement of the objective(s) or question(s) the review addresses. | 5 |
| **METHODS** | | |  |
| Eligibility criteria | 5 | Specify the inclusion and exclusion criteria for the review and how studies were grouped for the syntheses. | 6 |
| Information sources | 6 | Specify all databases, registers, websites, organisations, reference lists and other sources searched or consulted to identify studies. Specify the date when each source was last searched or consulted. | 6-7 |
| Search strategy | 7 | Present the full search strategies for all databases, registers and websites, including any filters and limits used. | Supplementary materials |
| Selection process | 8 | Specify the methods used to decide whether a study met the inclusion criteria of the review, including how many reviewers screened each record and each report retrieved, whether they worked independently, and if applicable, details of automation tools used in the process. | 7 |
| Data collection process | 9 | Specify the methods used to collect data from reports, including how many reviewers collected data from each report, whether they worked independently, any processes for obtaining or confirming data from study investigators, and if applicable, details of automation tools used in the process. | 7 |
| Data items | 10a | List and define all outcomes for which data were sought. Specify whether all results that were compatible with each outcome domain in each study were sought (e.g. for all measures, time points, analyses), and if not, the methods used to decide which results to collect. | 7 |
|  | 10b | List and define all other variables for which data were sought (e.g. participant and intervention characteristics, funding sources). Describe any assumptions made about any missing or unclear information. | 8 |
| Study risk of bias assessment | 11 | Specify the methods used to assess risk of bias in the included studies, including details of the tool(s) used, how many reviewers assessed each study and whether they worked independently, and if applicable, details of automation tools used in the process. | 7 |
| Effect measures | 12 | Specify for each outcome the effect measure(s) (e.g. risk ratio, mean difference) used in the synthesis or presentation of results. | 8 |
| Synthesis methods | 13a | Describe the processes used to decide which studies were eligible for each synthesis (e.g. tabulating the study intervention characteristics and comparing against the planned groups for each synthesis (item #5)). | 8 |
|  | 13b | Describe any methods required to prepare the data for presentation or synthesis, such as handling of missing summary statistics, or data conversions. | 8 |
|  | 13c | Describe any methods used to tabulate or visually display results of individual studies and syntheses. | 8 |
|  | 13d | Describe any methods used to synthesize results and provide a rationale for the choice(s). If meta-analysis was performed, describe the model(s), method(s) to identify the presence and extent of statistical heterogeneity, and software package(s) used. | 8 |
|  | 13e | Describe any methods used to explore possible causes of heterogeneity among study results (e.g. subgroup analysis, meta-regression). | 8 |
|  | 13f | Describe any sensitivity analyses conducted to assess robustness of the synthesized results. | 8 |
| Reporting bias assessment | 14 | Describe any methods used to assess risk of bias due to missing results in a synthesis (arising from reporting biases). | 7 |
| Certainty assessment | 15 | Describe any methods used to assess certainty (or confidence) in the body of evidence for an outcome. | 8 |
| **RESULTS** | | |  |
| Study selection | 16a | Describe the results of the search and selection process, from the number of records identified in the search to the number of studies included in the review, ideally using a flow diagram. | 9 |
|  | 16b | Cite studies that might appear to meet the inclusion criteria, but which were excluded, and explain why they were excluded. |  |
| Study characteristics | 17 | Cite each included study and present its characteristics. | 9 |
| Risk of bias in studies | 18 | Present assessments of risk of bias for each included study. | Supplementary materials |
| Results of individual studies | 19 | For all outcomes, present, for each study: (a) summary statistics for each group (where appropriate) and (b) an effect estimate and its precision (e.g. confidence/credible interval), ideally using structured tables or plots. |  |
| Results of syntheses | 20a | For each synthesis, briefly summarise the characteristics and risk of bias among contributing studies. | 9 |
|  | 20b | Present results of all statistical syntheses conducted. If meta-analysis was done, present for each the summary estimate and its precision (e.g. confidence/credible interval) and measures of statistical heterogeneity. If comparing groups, describe the direction of the effect. | 9 |
|  | 20c | Present results of all investigations of possible causes of heterogeneity among study results. | 9 |
|  | 20d | Present results of all sensitivity analyses conducted to assess the robustness of the synthesized results. |  |
| Reporting biases | 21 | Present assessments of risk of bias due to missing results (arising from reporting biases) for each synthesis assessed. | 11 |
| Certainty of evidence | 22 | Present assessments of certainty (or confidence) in the body of evidence for each outcome assessed. | 10 |
| **DISCUSSION** | | |  |
| Discussion | 23a | Provide a general interpretation of the results in the context of other evidence. | 11-13 |
|  | 23b | Discuss any limitations of the evidence included in the review. | 14 |
|  | 23c | Discuss any limitations of the review processes used. | 14 |
|  | 23d | Discuss implications of the results for practice, policy, and future research. | 14 |
| **OTHER INFORMATION** | | |  |
| Registration and protocol | 24a | Provide registration information for the review, including register name and registration number, or state that the review was not registered. | 3 |
|  | 24b | Indicate where the review protocol can be accessed, or state that a protocol was not prepared. | 15 |
|  | 24c | Describe and explain any amendments to information provided at registration or in the protocol. |  |
| Support | 25 | Describe sources of financial or non-financial support for the review, and the role of the funders or sponsors in the review. | 15 |
| Competing interests | 26 | Declare any competing interests of review authors. | 15 |
| Availability of data, code and other materials | 27 | Report which of the following are publicly available and where they can be found: template data collection forms; data extracted from included studies; data used for all analyses; analytic code; any other materials used in the review. | Supplementary materials |

*From:*  Page MJ, McKenzie JE, Bossuyt PM, Boutron I, Hoffmann TC, Mulrow CD, et al. The PRISMA 2020 statement: an updated guideline for reporting systematic reviews. BMJ 2021;372:n71. doi: 10.1136/bmj.n71

For more information, visit: <http://www.prisma-statement.org/>

# Reference:

1. Kuwabara K, Hirose M, Kato K, et al. Serological analysis of sensitization in allergic bronchopulmonary aspergillosis: a study on allergen components and interspecies relationships. *J Asthma*. Jun 2020;57(6):610-617. doi:10.1080/02770903.2019.1599387

2. Braun JJ, Pauli G, Schultz P, Gentine A, Ebbo D, de Blay F. Allergic fungal sinusitis associated with allergic bronchopulmonary aspergillosis: an uncommon sinobronchial allergic mycosis. *Am J Rhinol*. Jul-Aug 2007;21(4):412-6. doi:10.2500/ajr.2007.21.3051

3. Hsiao CW, Yen TH, Wu YC, et al. Comparison of Aspergillus-specific antibody cut-offs for the diagnosis of aspergillosis. *Front Microbiol*. 2022;13:1060727. doi:10.3389/fmicb.2022.1060727

4. Fricker-Hidalgo H, Coltey B, Llerena C, et al. Recombinant allergens combined with biological markers in the diagnosis of allergic bronchopulmonary aspergillosis in cystic fibrosis patients. *Clin Vaccine Immunol*. Sep 2010;17(9):1330-6. doi:10.1128/CVI.00200-10

5. Latzin P, Hartl D, Regamey N, Frey U, Schoeni MH, Casaulta C. Comparison of serum markers for allergic bronchopulmonary aspergillosis in cystic fibrosis. *Eur Respir J*. Jan 2008;31(1):36-42. doi:10.1183/09031936.00078107

6. Maleki M, Mortezaee V, Hassanzad M, et al. Prevalence of allergic bronchopulmonary aspergillosis in cystic fibrosis patients using two different diagnostic criteria. *Eur Ann Allergy Clin Immunol*. May 2020;52(3):104-111. doi:10.23822/EurAnnACI.1764-1489.121

7. Barrera C, Richaud-Thiriez B, Rocchi S, et al. New Commercially Available IgG Kits and Time-Resolved Fluorometric IgE Assay for Diagnosis of Allergic Bronchopulmonary Aspergillosis in Patients with Cystic Fibrosis. *Clin Vaccine Immunol*. Dec 23 2015;23(3):196-203. doi:10.1128/CVI.00498-15

8. Wang JL, Patterson R, Rosenberg M, Roberts M, Cooper BJ. Serum IgE and IgG antibody activity against Aspergillus fumigatus as a diagnostic aid in allergic bronchopulmonary aspergillosis. *Am Rev Respir Dis*. May 1978;117(5):917-27. doi:10.1164/arrd.1978.117.5.917

9. Saxena P, Choudhary H, Muthu V, et al. Which Are the Optimal Criteria for the Diagnosis of Allergic Bronchopulmonary Aspergillosis? A Latent Class Analysis. *J Allergy Clin Immunol Pract*. Jan 2021;9(1):328-335.e1. doi:10.1016/j.jaip.2020.08.043

10. Mortezaee V, Mahdaviani SA, Pourabdollah M, et al. Diagnosis of allergic bronchopulmonary aspergillosis in patients with persistent allergic asthma using three different diagnostic algorithms. *Mycoses*. Mar 2021;64(3):272-281. doi:10.1111/myc.13217

11. Brummund W, Resnick A, Fink JN, Kurup VP. Aspergillus fumigatus-specific antibodies in allergic bronchopulmonary aspergillosis and aspergilloma: evidence for a polyclonal antibody response. *J Clin Microbiol*. Jan 1987;25(1):5-9. doi:10.1128/jcm.25.1.5-9.1987
