# Supplementary figures and images for: Comparison of diagnostic efficiency of detecting IgG and IgE with immunoassay method in diagnosing ABPA: a meta-analysis
Source: BMC Pulm Med. 2023 Oct 5;23:374. doi: 10.1186/s12890-023-02620-3 (PMC10557217; doi:10.1186/s12890-023-02620-3)

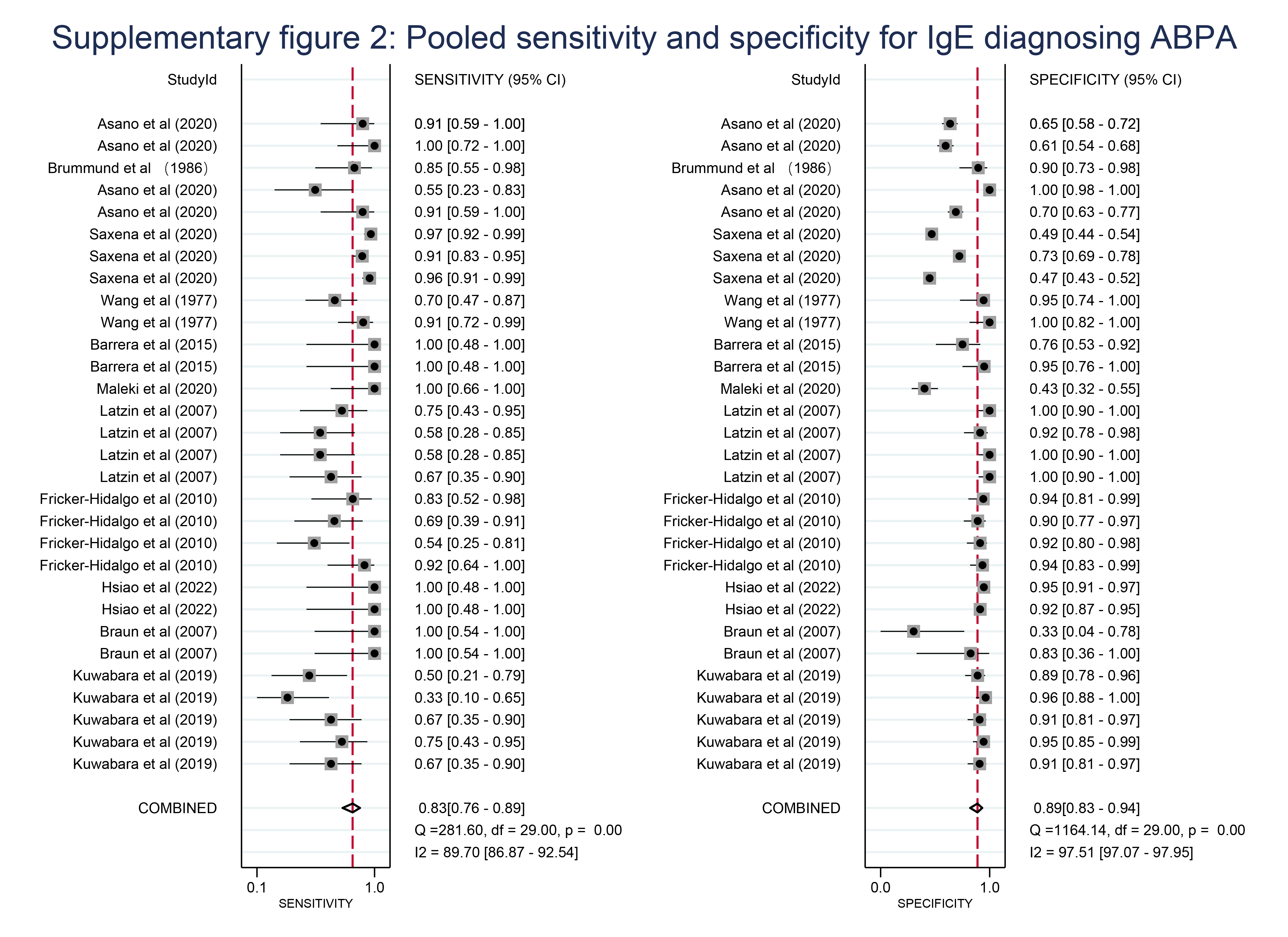

Supplement: Supplementary file 1 — Additional file 1: Supplementary Table 1. Detailed information of included studies. Supplementary Table 2. Summary performance for IgE and IgG in diagnosing ABPA. Supplementary Figure 1. Risk of bias and applicability concerns summary. Supplementary Figure 2. Forest plot of pooled sensitivity and specificity of the included articles (n = 12). Supplementary Figure 3. Forest plot of positive likelihood ratio and negative likelihood ratio of the included articles (n = 12). Supplementary Figure 4. Forest plot of the diagnostic score and diagnostic odds ratio of the included articles (n = 12). Supplementary Figure 5. Forest plot of pooled sensitivity and specificity of the included articles (n = 12). Supplementary Figure 6. Forest plot of positive likelihood ratio and negative likelihood ratio of the included articles (n = 12). Supplementary Figure 7. Forest plot of the diagnostic score and diagnostic odds ratio of the included articles (n = 12). Supplementary Figure 8. Sensitivity analysis of IgE and IgG (n = 12). [file 12890_2023_2620_MOESM1_ESM.zip › 12890_2023_2620_MOESM2_ESM.tif]

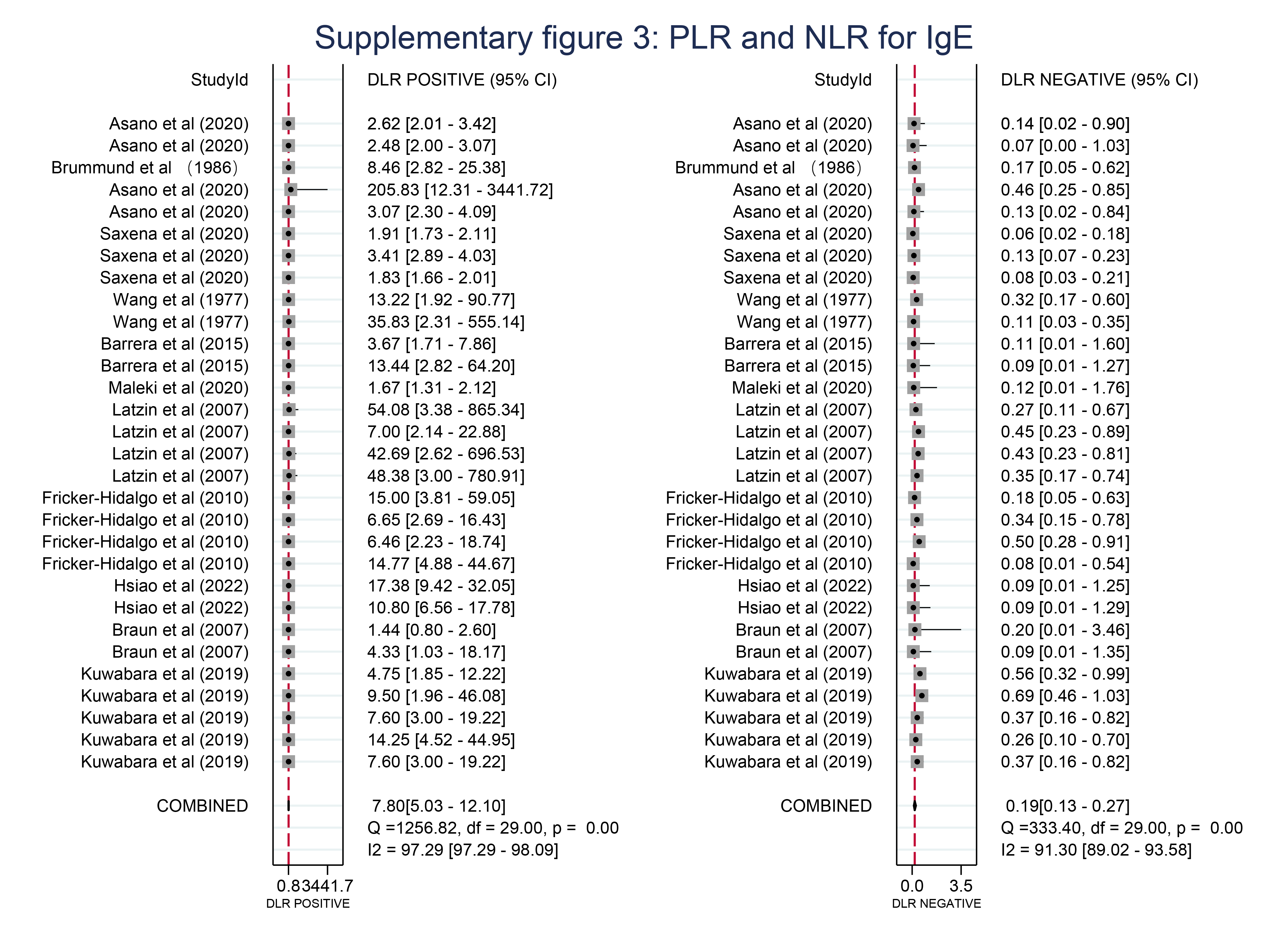

Supplement: Supplementary file 1 — Additional file 1: Supplementary Table 1. Detailed information of included studies. Supplementary Table 2. Summary performance for IgE and IgG in diagnosing ABPA. Supplementary Figure 1. Risk of bias and applicability concerns summary. Supplementary Figure 2. Forest plot of pooled sensitivity and specificity of the included articles (n = 12). Supplementary Figure 3. Forest plot of positive likelihood ratio and negative likelihood ratio of the included articles (n = 12). Supplementary Figure 4. Forest plot of the diagnostic score and diagnostic odds ratio of the included articles (n = 12). Supplementary Figure 5. Forest plot of pooled sensitivity and specificity of the included articles (n = 12). Supplementary Figure 6. Forest plot of positive likelihood ratio and negative likelihood ratio of the included articles (n = 12). Supplementary Figure 7. Forest plot of the diagnostic score and diagnostic odds ratio of the included articles (n = 12). Supplementary Figure 8. Sensitivity analysis of IgE and IgG (n = 12). [file 12890_2023_2620_MOESM1_ESM.zip › 12890_2023_2620_MOESM3_ESM.tif]

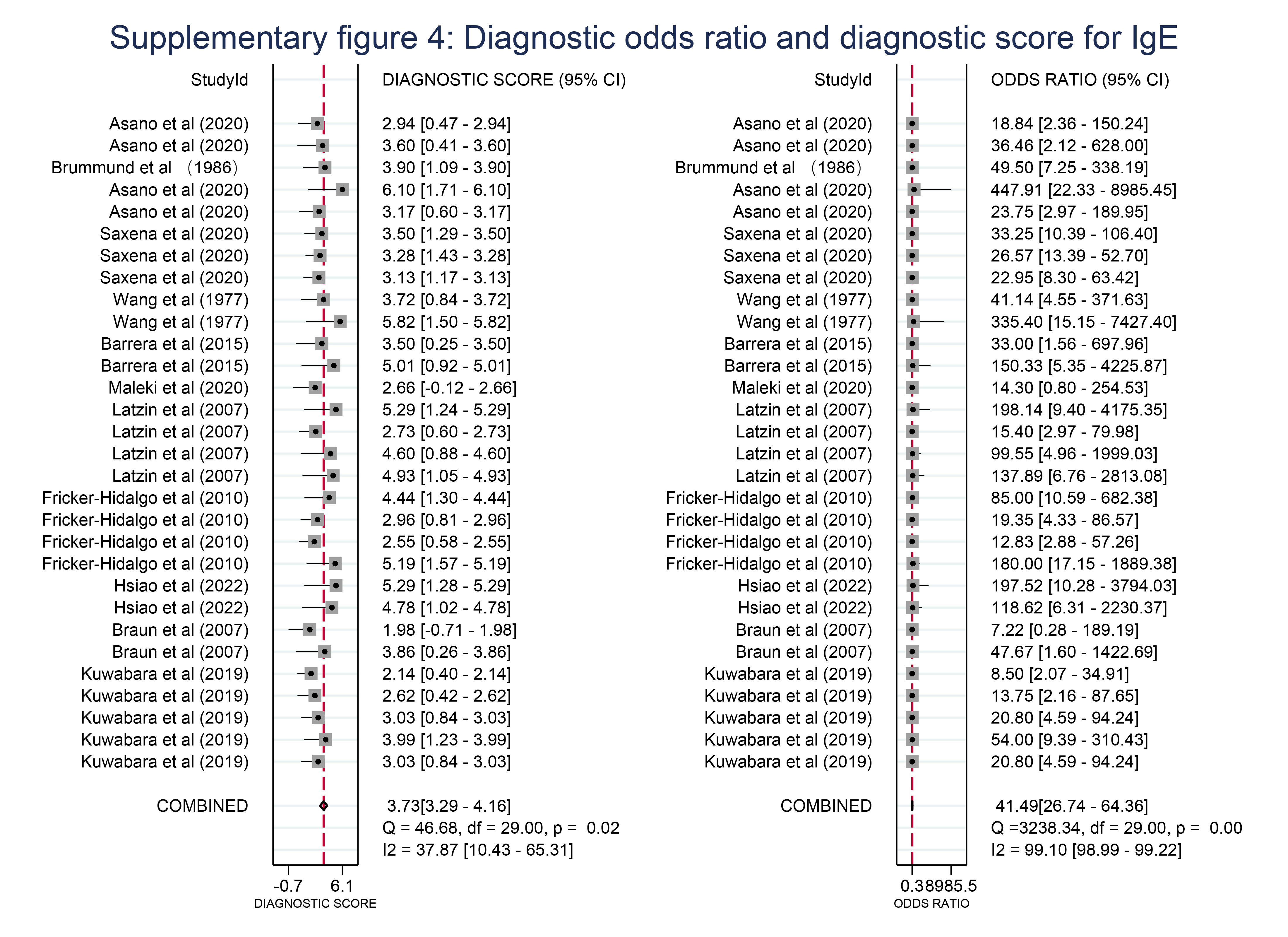

Supplement: Supplementary file 1 — Additional file 1: Supplementary Table 1. Detailed information of included studies. Supplementary Table 2. Summary performance for IgE and IgG in diagnosing ABPA. Supplementary Figure 1. Risk of bias and applicability concerns summary. Supplementary Figure 2. Forest plot of pooled sensitivity and specificity of the included articles (n = 12). Supplementary Figure 3. Forest plot of positive likelihood ratio and negative likelihood ratio of the included articles (n = 12). Supplementary Figure 4. Forest plot of the diagnostic score and diagnostic odds ratio of the included articles (n = 12). Supplementary Figure 5. Forest plot of pooled sensitivity and specificity of the included articles (n = 12). Supplementary Figure 6. Forest plot of positive likelihood ratio and negative likelihood ratio of the included articles (n = 12). Supplementary Figure 7. Forest plot of the diagnostic score and diagnostic odds ratio of the included articles (n = 12). Supplementary Figure 8. Sensitivity analysis of IgE and IgG (n = 12). [file 12890_2023_2620_MOESM1_ESM.zip › 12890_2023_2620_MOESM4_ESM.tif]

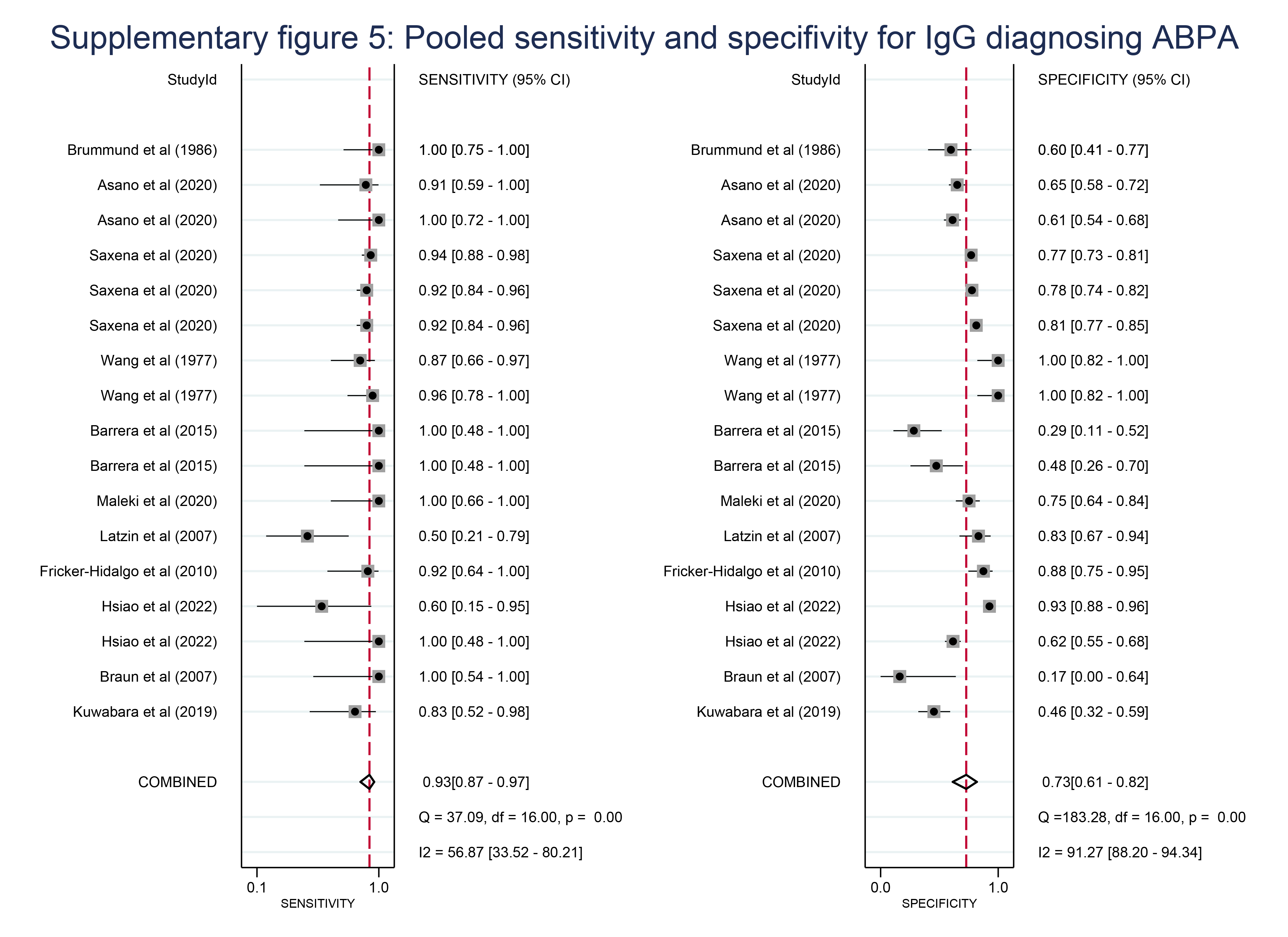

Supplement: Supplementary file 1 — Additional file 1: Supplementary Table 1. Detailed information of included studies. Supplementary Table 2. Summary performance for IgE and IgG in diagnosing ABPA. Supplementary Figure 1. Risk of bias and applicability concerns summary. Supplementary Figure 2. Forest plot of pooled sensitivity and specificity of the included articles (n = 12). Supplementary Figure 3. Forest plot of positive likelihood ratio and negative likelihood ratio of the included articles (n = 12). Supplementary Figure 4. Forest plot of the diagnostic score and diagnostic odds ratio of the included articles (n = 12). Supplementary Figure 5. Forest plot of pooled sensitivity and specificity of the included articles (n = 12). Supplementary Figure 6. Forest plot of positive likelihood ratio and negative likelihood ratio of the included articles (n = 12). Supplementary Figure 7. Forest plot of the diagnostic score and diagnostic odds ratio of the included articles (n = 12). Supplementary Figure 8. Sensitivity analysis of IgE and IgG (n = 12). [file 12890_2023_2620_MOESM1_ESM.zip › 12890_2023_2620_MOESM5_ESM.tif]

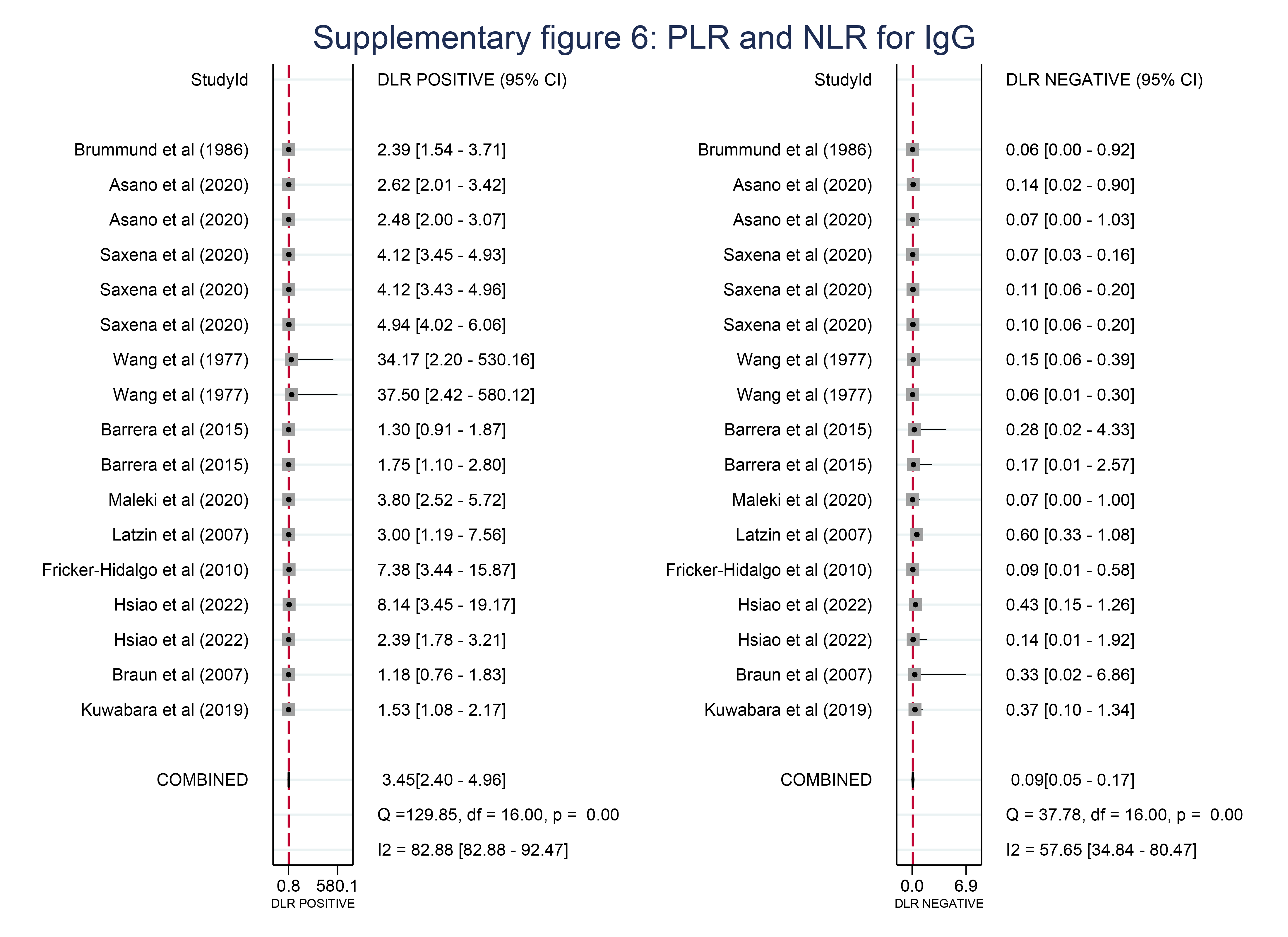

Supplement: Supplementary file 1 — Additional file 1: Supplementary Table 1. Detailed information of included studies. Supplementary Table 2. Summary performance for IgE and IgG in diagnosing ABPA. Supplementary Figure 1. Risk of bias and applicability concerns summary. Supplementary Figure 2. Forest plot of pooled sensitivity and specificity of the included articles (n = 12). Supplementary Figure 3. Forest plot of positive likelihood ratio and negative likelihood ratio of the included articles (n = 12). Supplementary Figure 4. Forest plot of the diagnostic score and diagnostic odds ratio of the included articles (n = 12). Supplementary Figure 5. Forest plot of pooled sensitivity and specificity of the included articles (n = 12). Supplementary Figure 6. Forest plot of positive likelihood ratio and negative likelihood ratio of the included articles (n = 12). Supplementary Figure 7. Forest plot of the diagnostic score and diagnostic odds ratio of the included articles (n = 12). Supplementary Figure 8. Sensitivity analysis of IgE and IgG (n = 12). [file 12890_2023_2620_MOESM1_ESM.zip › 12890_2023_2620_MOESM6_ESM.tif]

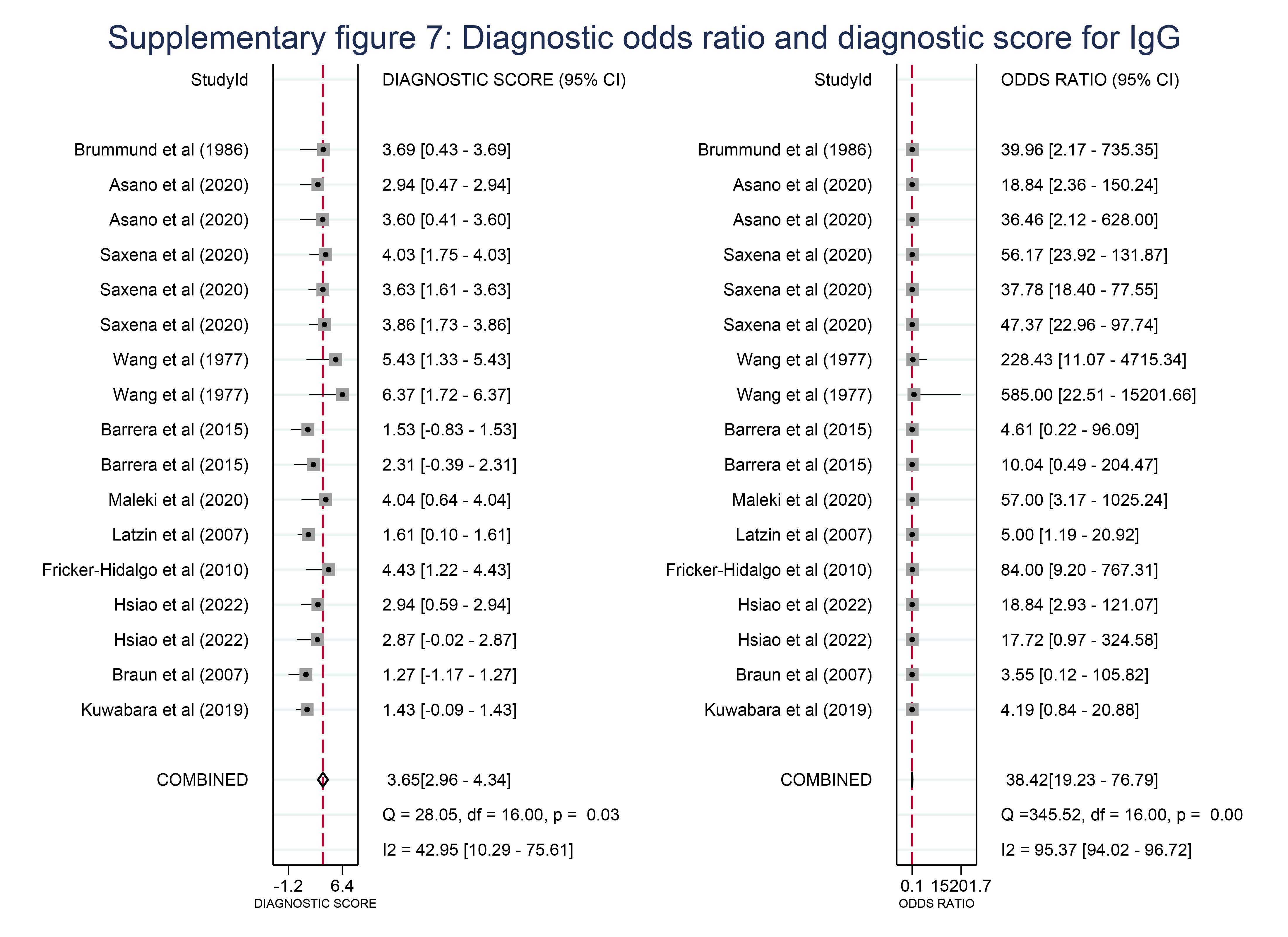

Supplement: Supplementary file 1 — Additional file 1: Supplementary Table 1. Detailed information of included studies. Supplementary Table 2. Summary performance for IgE and IgG in diagnosing ABPA. Supplementary Figure 1. Risk of bias and applicability concerns summary. Supplementary Figure 2. Forest plot of pooled sensitivity and specificity of the included articles (n = 12). Supplementary Figure 3. Forest plot of positive likelihood ratio and negative likelihood ratio of the included articles (n = 12). Supplementary Figure 4. Forest plot of the diagnostic score and diagnostic odds ratio of the included articles (n = 12). Supplementary Figure 5. Forest plot of pooled sensitivity and specificity of the included articles (n = 12). Supplementary Figure 6. Forest plot of positive likelihood ratio and negative likelihood ratio of the included articles (n = 12). Supplementary Figure 7. Forest plot of the diagnostic score and diagnostic odds ratio of the included articles (n = 12). Supplementary Figure 8. Sensitivity analysis of IgE and IgG (n = 12). [file 12890_2023_2620_MOESM1_ESM.zip › 12890_2023_2620_MOESM7_ESM.tif]

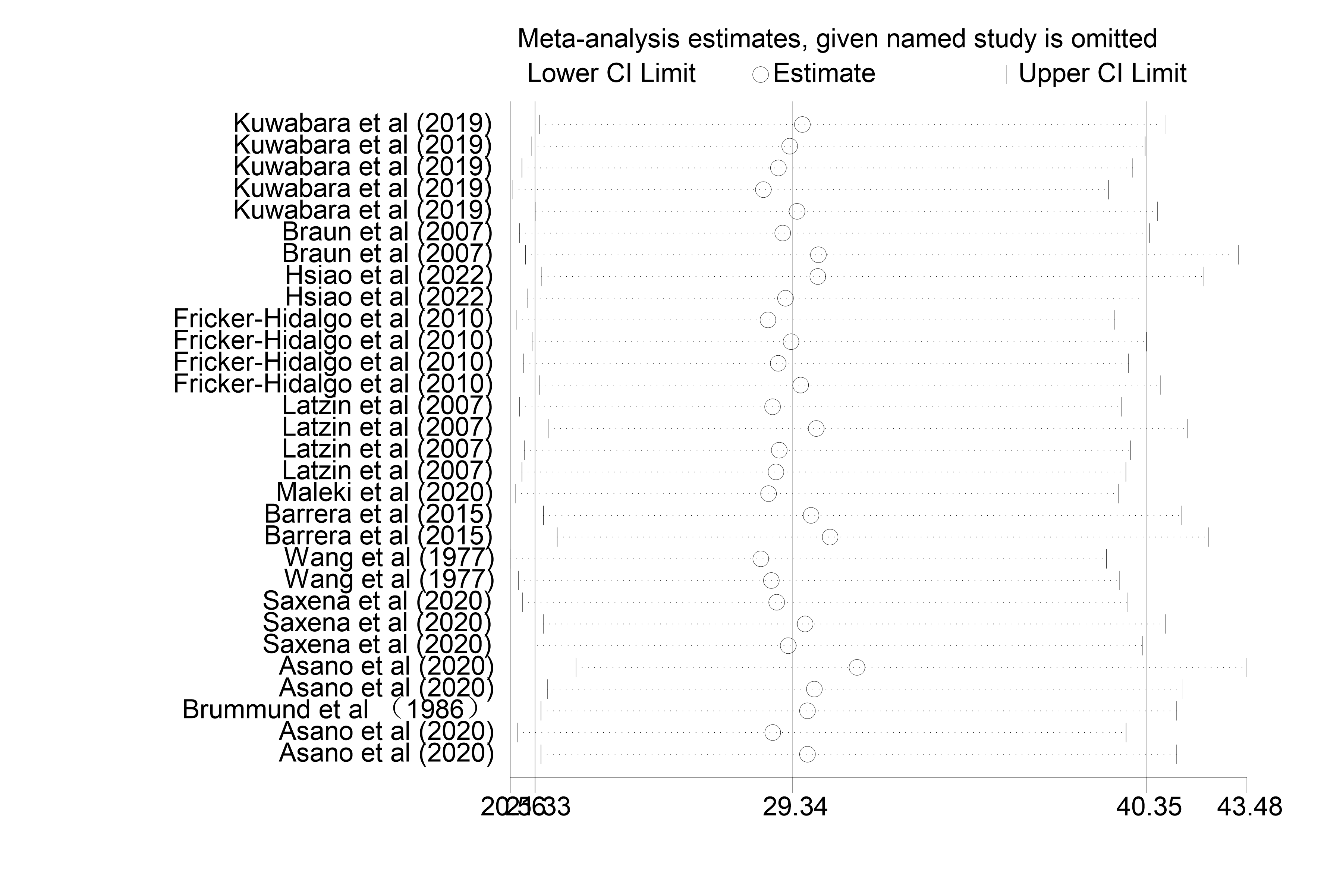

Supplement: Supplementary file 1 — Additional file 1: Supplementary Table 1. Detailed information of included studies. Supplementary Table 2. Summary performance for IgE and IgG in diagnosing ABPA. Supplementary Figure 1. Risk of bias and applicability concerns summary. Supplementary Figure 2. Forest plot of pooled sensitivity and specificity of the included articles (n = 12). Supplementary Figure 3. Forest plot of positive likelihood ratio and negative likelihood ratio of the included articles (n = 12). Supplementary Figure 4. Forest plot of the diagnostic score and diagnostic odds ratio of the included articles (n = 12). Supplementary Figure 5. Forest plot of pooled sensitivity and specificity of the included articles (n = 12). Supplementary Figure 6. Forest plot of positive likelihood ratio and negative likelihood ratio of the included articles (n = 12). Supplementary Figure 7. Forest plot of the diagnostic score and diagnostic odds ratio of the included articles (n = 12). Supplementary Figure 8. Sensitivity analysis of IgE and IgG (n = 12). [file 12890_2023_2620_MOESM1_ESM.zip › 12890_2023_2620_MOESM8_ESM.tif]

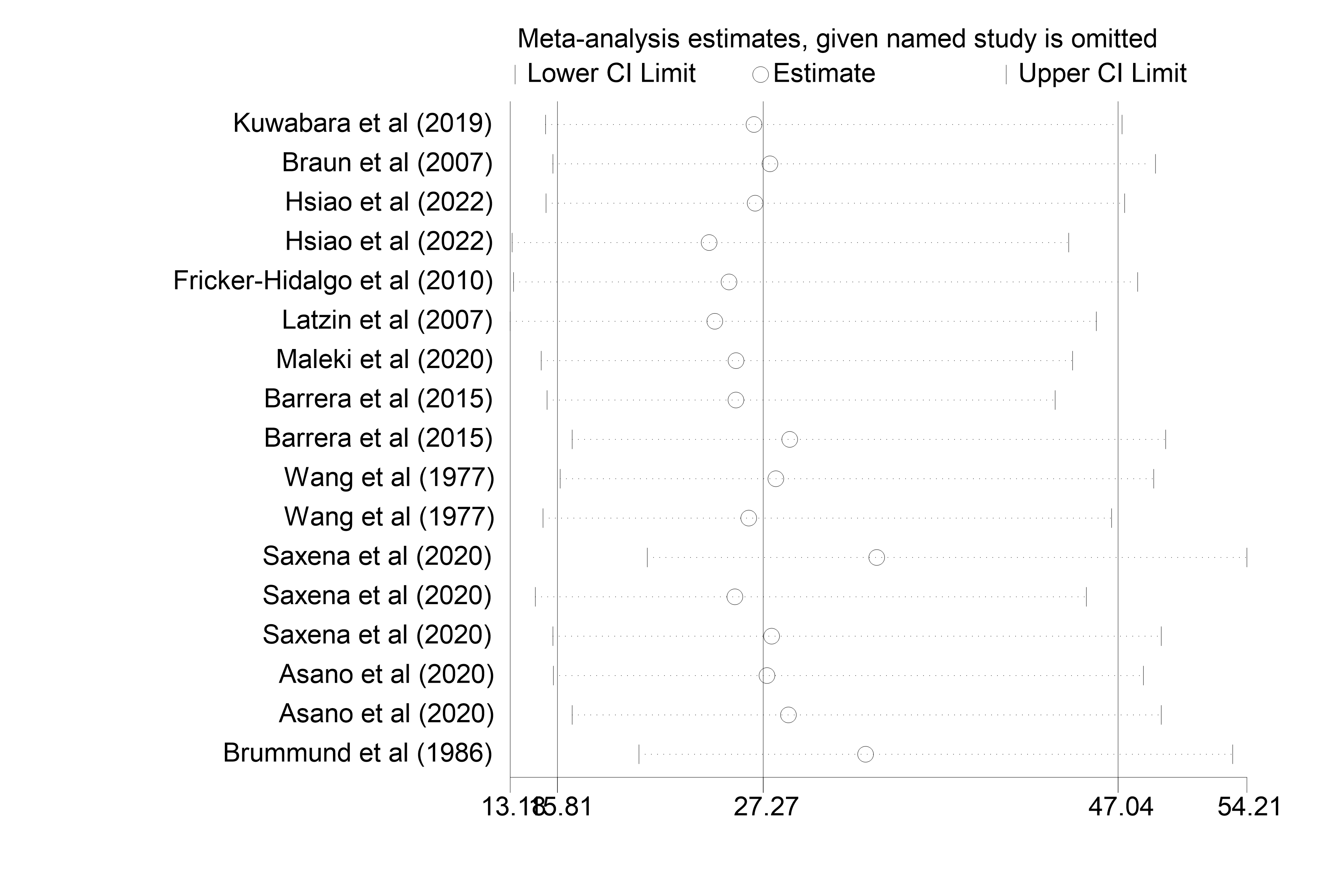

Supplement: Supplementary file 1 — Additional file 1: Supplementary Table 1. Detailed information of included studies. Supplementary Table 2. Summary performance for IgE and IgG in diagnosing ABPA. Supplementary Figure 1. Risk of bias and applicability concerns summary. Supplementary Figure 2. Forest plot of pooled sensitivity and specificity of the included articles (n = 12). Supplementary Figure 3. Forest plot of positive likelihood ratio and negative likelihood ratio of the included articles (n = 12). Supplementary Figure 4. Forest plot of the diagnostic score and diagnostic odds ratio of the included articles (n = 12). Supplementary Figure 5. Forest plot of pooled sensitivity and specificity of the included articles (n = 12). Supplementary Figure 6. Forest plot of positive likelihood ratio and negative likelihood ratio of the included articles (n = 12). Supplementary Figure 7. Forest plot of the diagnostic score and diagnostic odds ratio of the included articles (n = 12). Supplementary Figure 8. Sensitivity analysis of IgE and IgG (n = 12). [file 12890_2023_2620_MOESM1_ESM.zip › 12890_2023_2620_MOESM9_ESM.tif]
